# Supplementary material for: A murine model lacking Lyst recapitulates Chediak-Higashi syndrome with an earlier-onset neurodegenerative phenotype
Source: Commun Biol. 2025 Jul 18;8:1064. doi: 10.1038/s42003-025-08482-1 (PMC12274407; doi:10.1038/s42003-025-08482-1)
Supplement: Supplementary file 1 — Supplementary Information [file 42003_2025_8482_MOESM1_ESM.pdf]

Supplementary Information

Supplementary Figures and Legends

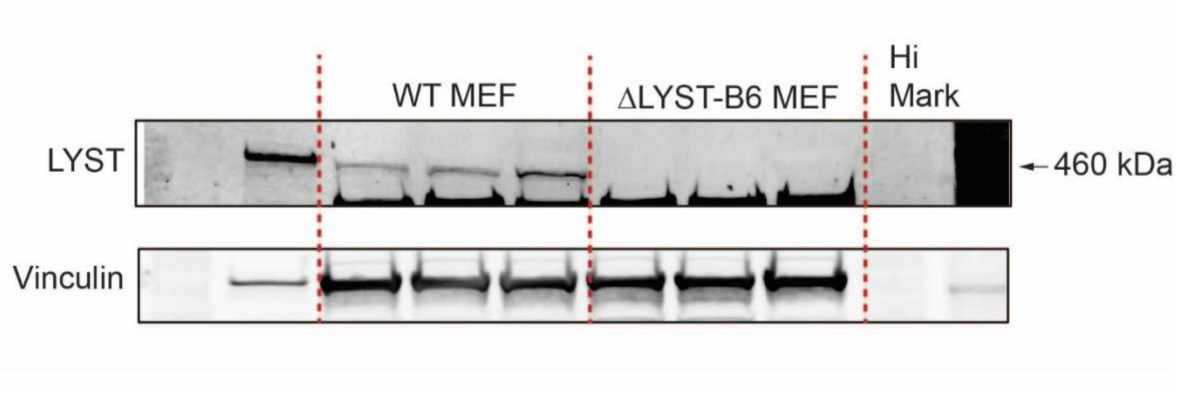

Supplementary Figure 1. LYST protein levels detected by western blot in WT and ΔLYST-B6 mouse embryonic fibroblasts (MEFs) relative to the vinculin loading control (two biological replicates- first two bands- with one technical replicate in the third well). WT, wild-type; ΔLYST-B6, *Lyst* homozygous knockout (C57BL/6J background).

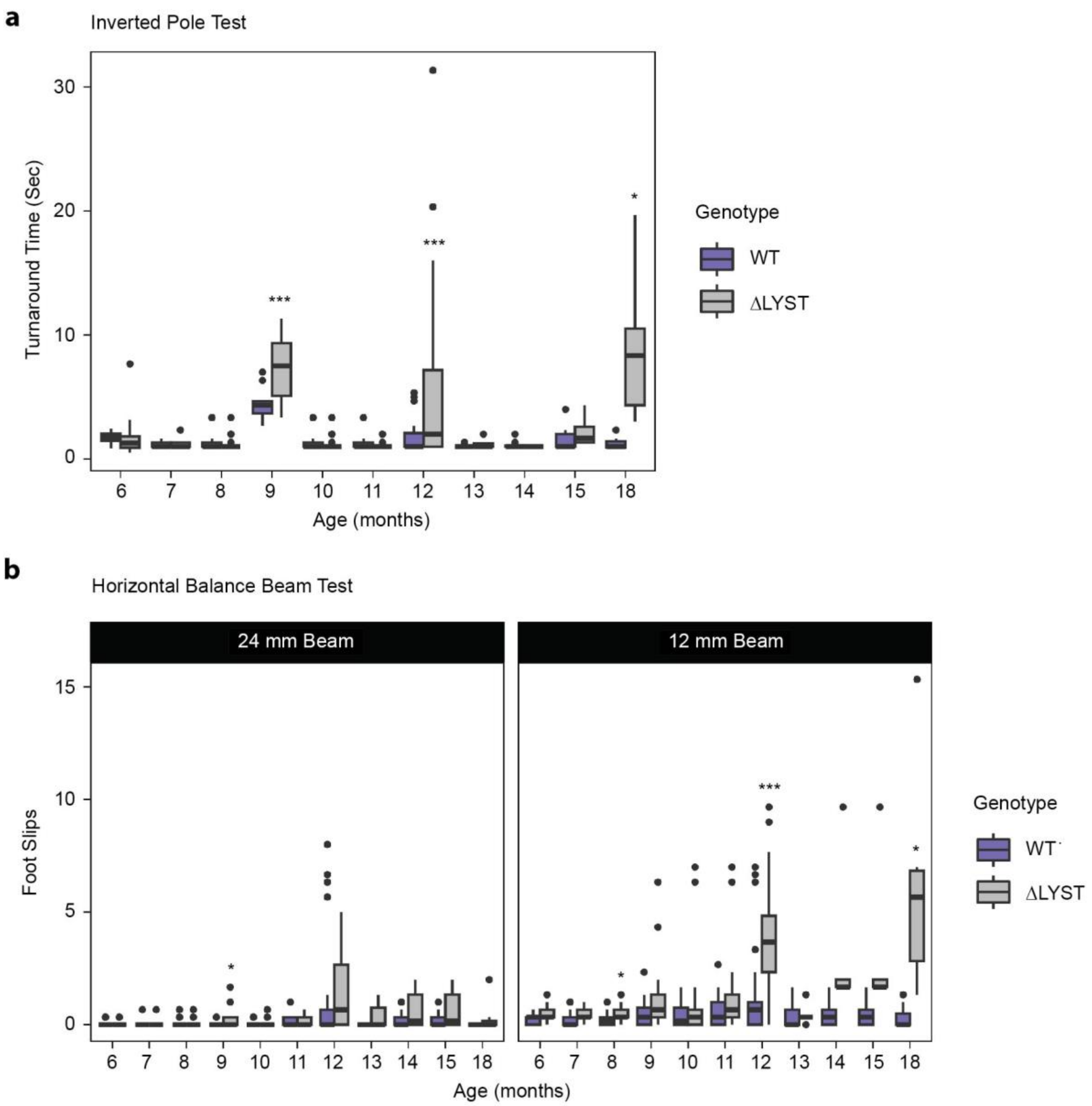

Supplementary Figure 2. Additional neurobehavioral analysis of the ΔLYST-B6 mouse model. **a**. Average turnaround times from the inverted pole test of WT and ΔLYST-B6 mice from the ages of 6 to 18 months. **b.** Average number of foot slips of WT and ΔLYST-B6 mice while crossing a 24-mm beam and 12-mm beam. WT, wild-type; ΔLYST-B6, *Lyst* homozygous knockout (C57BL/6J background). Student's two-sample t-test: * = P<0.05, ** = P<0.01, *** = P<0.001.

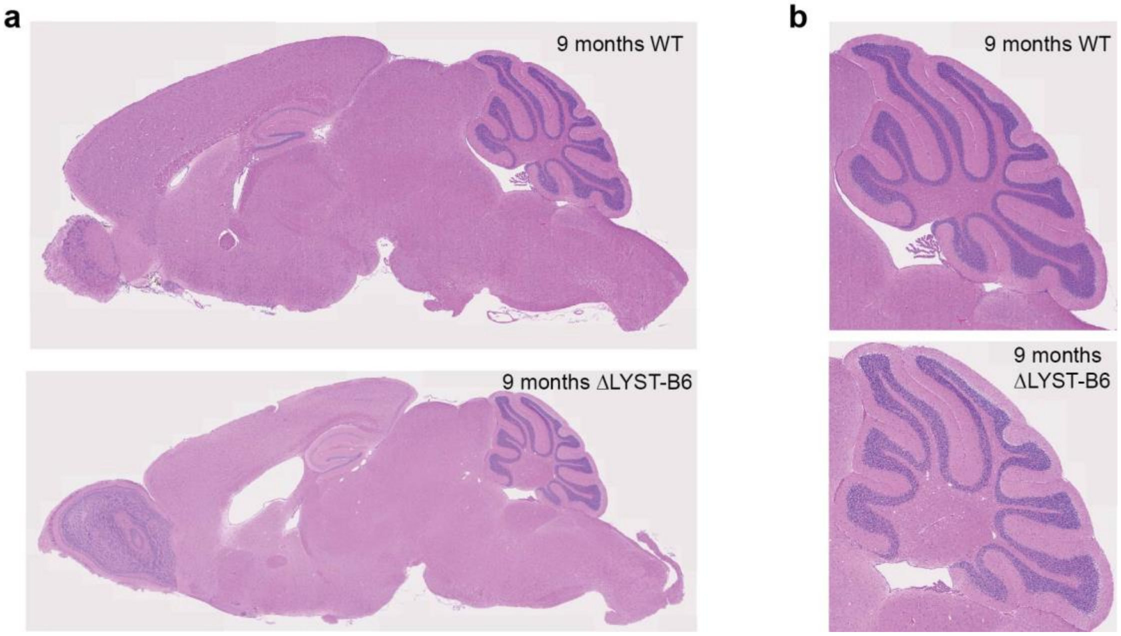

Supplementary Figure 3. a. Representative images of H&E-stained sagittal brain of 9-month-old WT control and ΔLYST-B6 mice. **b.** Representative images of H&E-stained cerebellum of 9-month-old WT control and ΔLYST-B6 mice Purkinje cells are dark purple/blue cells above the purple granular layer. ΔLYST-B6 mice showed a reduction in the Purkinje cell layer. WT, wild-type; ΔLYST-B6, *Lyst* homozygous knockout (C57BL/6J background).

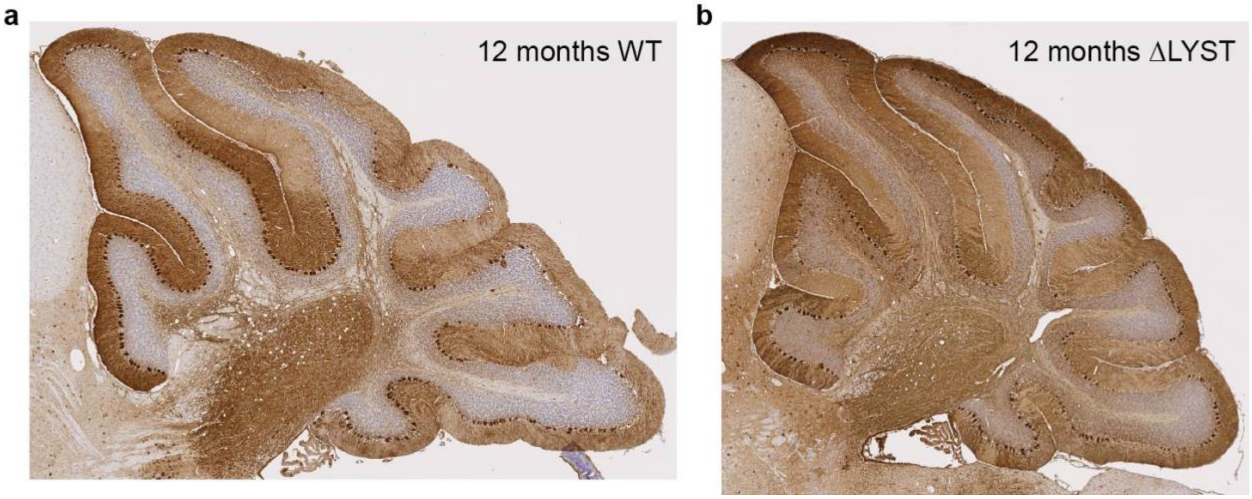

Supplementary Figure 4. Representative images of Calbindin-28k stained cerebellum of 12-month-old WT control (**a**) and ΔLYST-B6 mice (**b**). Purkinje cells are dark brown cells above the

blue-grey granular layer. ΔLYST-B6 mice showed a reduction in the Purkinje cell layer. WT, wild-type; ΔLYST-B6, *Lyst* homozygous knockout (C57BL/6J background).

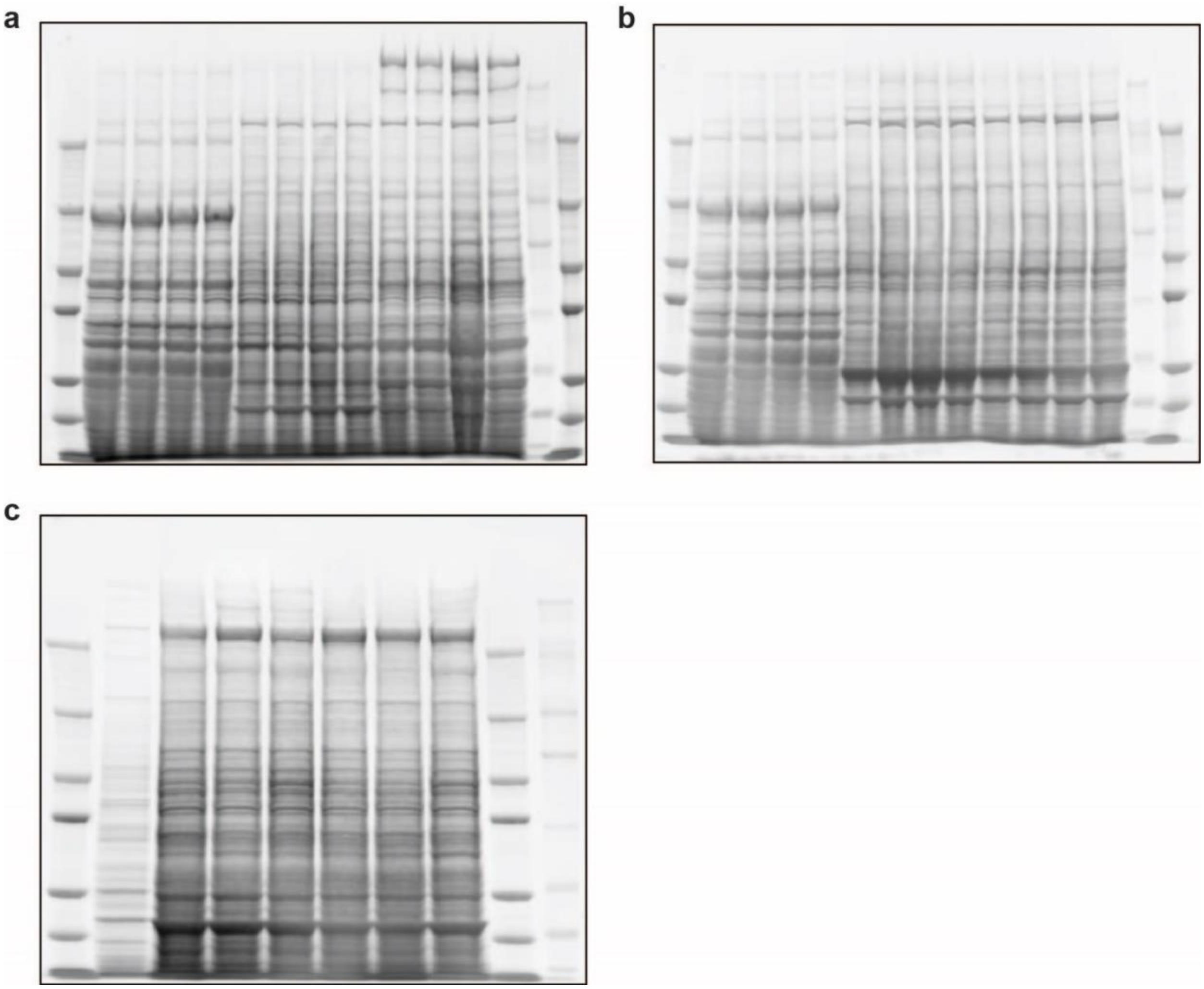

Supplementary Figure 5. All uncropped western blots used in this study. Western blots in the main figures are cropped to show bands of interest for LYST and vinculin control. Here we show the full blots from Figure 1d (**a, b**) and Supplementary Figure 1 (**c**).

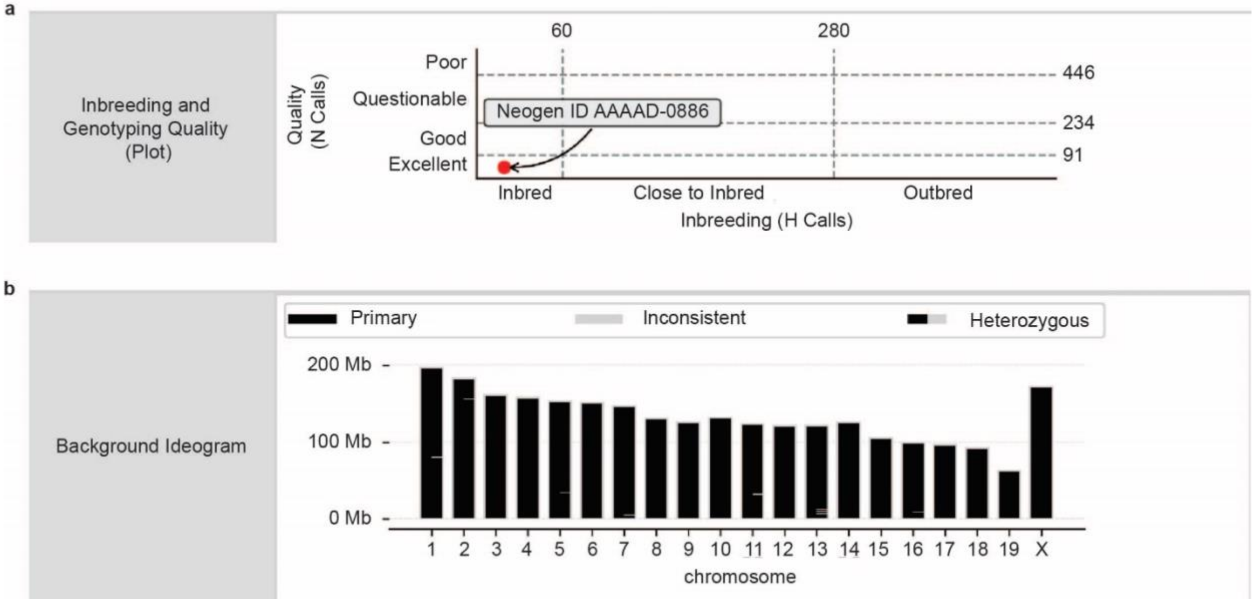

Supplementary Figure 6. SNP array showing complete backcrossing of the ΔLYST mutation on to a DBA/2J background (ΔLYST-DBA).

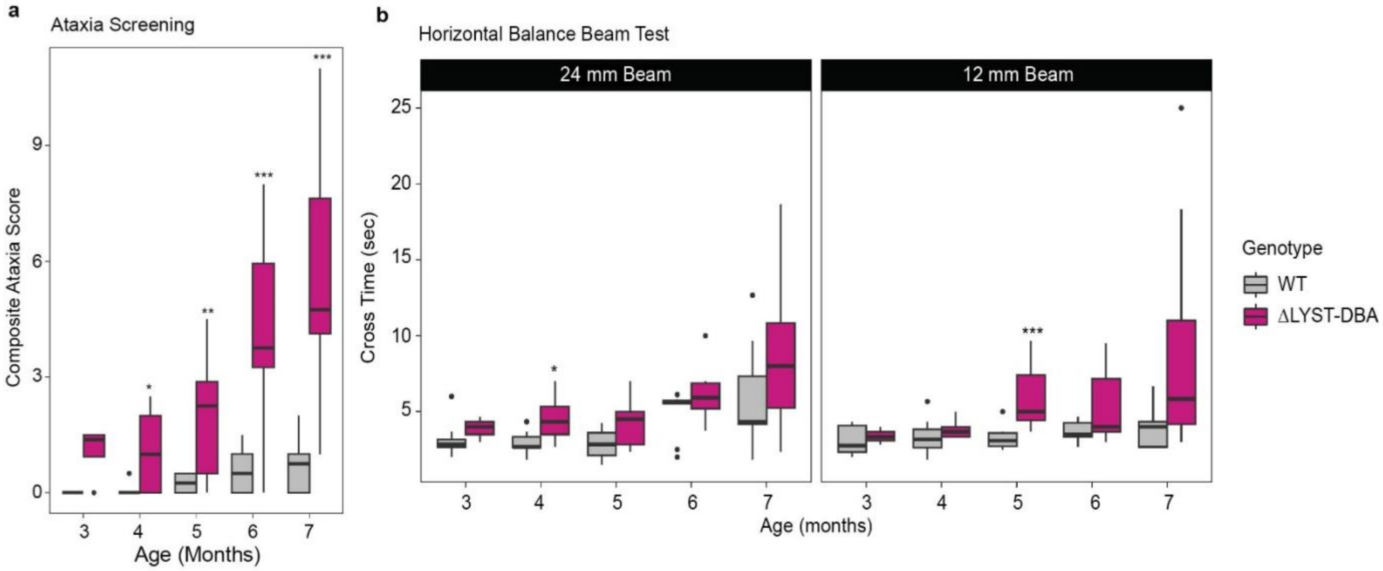

Supplementary Figure 7. Neurobehavioral analyses of the ΔLYST-DBA mouse model. **a.** Average cumulative ataxia scores from ataxia screening of WT and ΔLYST-DBA mice from the age of 3 to 7 months. **b.** Average cross time of 24-mm beam and 12-mm beam from the

horizontal balance beam test of WT and ΔLYST-DBA mice from the age of 3 months to 7 months. WT, wild-type; ΔLYST-DBA, *Lyst* homozygous knockout (DBA/2J background). Student's two-sample t-test: * = $P<0.05$, ** = $P<0.01$, *** = $P<0.001$.

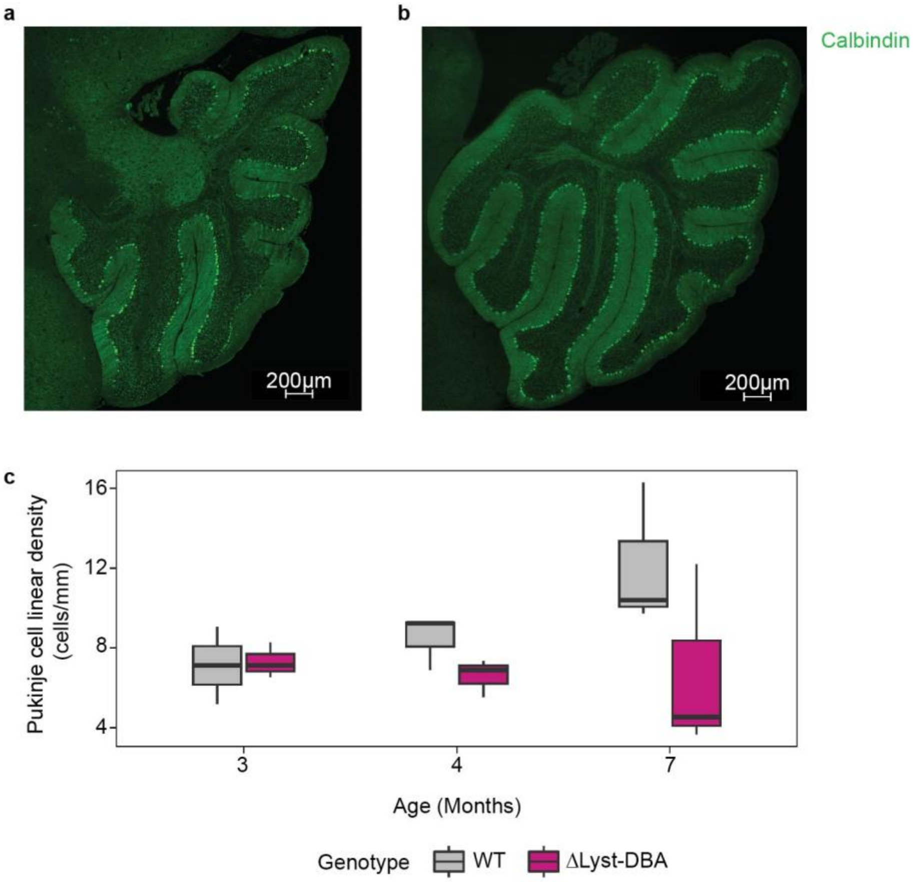

Supplementary Figure 8. Immunofluorescent analyses of ΔLYST-DBA cerebellum. **a.** Representative image of immunofluorescent Calbindin-28k stained cerebellum of 7-month-old WT control. **b.** Representative image of immunofluorescent Calbindin-28k stained cerebellum of 7-month-old ΔLYST-DBA mouse. Purkinje cells are the bright green cells above the blue layer. ΔLYST-DBA mice showed a loss of the Purkinje cell layer as compared to WT controls. **c.** Average number of Purkinje cells in 3-, 4-, and 7-month-old WT and ΔLYST-DBA mice. WT, wild-type; ΔLYST-DBA, *Lyst* homozygous knockout (DBA/2J background).

Supplementary Tables

Supplementary Table 1. Guide RNA, genotyping primers and PCR thermocycling parameters

Supplementary Table 1a. Guide RNA (5'-3')

Target Exon	Promoter	sgRNA Target Site	Universal Primer Overlap
Exon 4	taatacgactcactatag	gtggccctgctgttaacatg	ttttagagctaga
Exon 53	taatacgactcactatag	gtcactgctggtacaagtcg	ttttagagctaga

Supplementary Table 1b. Genotyping Primers

Primer Name	Direction	Primer Sequence (5'-3')
UpF	Forward	gcatttgctgctaagcctaacagtacattc
UpR	Reverse	gctattgctgggacacttgcgacat
DnR	Reverse	ggagcccttgctgacaggctttggcctattag

Supplementary Table 1c. Genotyping PCR Thermocycling Parameters

Temperature (°C)	Time	
95	3 min	
95	30 sec	
55	30 sec	30 cycles
72	1 min	
72	7 min	
10	∞	

Supplementar2 Table 2. Antibodies used

Supplementary Table 2a. Primary Antibodies

Antibody	Manufacturer	Catalog Number	Dilution
Anti-LYST rabbit polyclonal	Atlas antibodies	HPA055725	1:555
Anti-LYST rabbit polyclonal	Novus Biologicals	NBP2-56533	1:625
Anti-vinculin mouse monoclonal	Sigma	SAB4200080, clone V284, purified	1:10,000
Anti-LAMP1	Developmental Studies Hybridoma Bank	1D4B	1:200
Rabbit anti-Calbindin D-28k	Swant	CB38	1:10,000
Anti-PCP4	Thermo Scientific	PA5-52209	1:1000
Anti-GFAP	Invitrogen	PA1-10004	1:1000

Supplementary Table 2b. Secondary Antibodies

Antibody	Manufacturer	Catalog Number	Dilution
IRDYE 800CW-conjugated donkey anti-rabbit IgG	LI-COR Biosciences	926-32213	1:5000
IRDYE 680RD-conjugated donkey anti-mouse	LI-COR Biosciences	926-68072	1:5000
Alexa Fluor 488	Invitrogen	A21208	1:300
Alexa Fluor Phalloidin 555	Invitrogen	A34055	1:40
Alexa Fluor 633	Invitrogen	A11039	1:100
Alexa Fluor 555	Abcam	Ab150154	1:100
Alexa Fluor 488	Invitrogen	A21070	1:100
