# Supplementary material for: A murine model lacking Lyst recapitulates Chediak-Higashi syndrome with an earlier-onset neurodegenerative phenotype
Source: Commun Biol. 2025 Jul 18;8:1064. doi: 10.1038/s42003-025-08482-1 (PMC12274407; doi:10.1038/s42003-025-08482-1)
Supplement: Supplementary file 2 — Supplementary Data 1 [file 42003_2025_8482_MOESM2_ESM.docx]

**Supplementary Data 1.** Deleted allele sequence

Determined by UCSC mouse genome browser GRCm38/mm10

Genomic: 13,630,081-13,778,963: **148,883 bp deleted**

Exon 4 to Exon 53 deleted: 9001 bp deleted

Deleted sequence using PCR primers (UpF-DnR)

ATTAAATAAATAACATATAATTAAAAAGATTAAGTCACTCCATCATCTTTAGCCTAACTTAGTATCCCTCTTAGATTTTCTTTCTTGGTGGAGATATTTTTGGCACCTAGTTACCTAAGCCAGAAACCTGTGAGGAGTTTTTTCCTCACTTGTACATCATTGAAAATTAACTATGAGCCATTTCAGTTCTACT

GTCAGGACTCAAGATGAGACTGTATGGCTGCAGCCATGCAGATAAGCACAGATACCATGTGGCTAGAGAGAAGGAAACAATAGCCAATGGATTAGATTAAGAGCACCATGAATAGTAATAGCTCATGAGACAGGGCCTATGACAGTCTGCACCTACAGTTGGGCTTTATCACAGCTTAGTCAGGACAGCAGATGTGGGAAGAAACACAGAATTCAGCAGAGATCAGAGTCAGTAGTGCTCTGTGTAAGAATCCACAGTCCATTGAGTCTTCCCAGAAGCCAAGAAGCAGATGCCCTTACTAAAGTACAGGCCATCTGCCCTCGGGCACCCCAAGACTGCTGGATTCATTAGTGTGGCCACAGGAATTCAGTGTAGGCTGGAGAATAGCTGAATCA
