# Supplementary material for: A murine model lacking Lyst recapitulates Chediak-Higashi syndrome with an earlier-onset neurodegenerative phenotype
Source: Commun Biol. 2025 Jul 18;8:1064. doi: 10.1038/s42003-025-08482-1 (PMC12274407; doi:10.1038/s42003-025-08482-1)
Supplement: Supplementary file 7 — Supplementary Data 5 [file 42003_2025_8482_MOESM7_ESM.zip › B6 mice data/B6 HET Aging Study/CHS Het Aging Study Mice.docx]

| **Cage #** | **DOB** | **Age (Months)** | **Age (weeks)** | **EP #** | **sex** | **Mouse type** | **Parent CC** |
| --- | --- | --- | --- | --- | --- | --- | --- |
| 3308 | 7/7/21 | 19.80 | 86.00 | 2 | M | HET | 30283 |
| 3241 | 7/7/21 | 19.80 | 86.00 | 1 | F | HET | 30283 |
| 3241 | 7/7/21 | 19.80 | 86.00 | 2 | F | HET | 30283 |
| 3245 | 7/11/21 | 19.67 | 85.43 | 1 | F | HET | 3210 |
| 3245 | 7/11/21 | 19.67 | 85.43 | 2 | F | HET | 3210 |
| 19297 | 8/16/21 | 18.50 | 80.29 | 1 | M | HET | 30283 |
| 19297 | 8/16/21 | 18.50 | 80.29 | 2 | M | HET | 30283 |
| 19297 | 8/16/21 | 18.50 | 80.29 | 3 | M | HET | 30283 |
| 19297 | 8/16/21 | 18.50 | 80.29 | 4 | M | HET | 30283 |
| 19273 | 8/16/21 | 18.50 | 80.29 | 1 | F | HET | 30283 |
| 19273 | 8/16/21 | 18.50 | 80.29 | 2 | F | HET | 30283 |
| 19273 | 8/16/21 | 18.50 | 80.29 | 3 | F | HET | 30283 |
| 19273 | 8/16/21 | 18.50 | 80.29 | 4 | F | HET | 30283 |
| 19273 | 8/16/21 | 18.50 | 80.29 | 5 | F | HET | 30283 |
| 19242 | 8/23/21 | 18.27 | 79.29 | 1 | F | HET | 3210 |
| 19242 | 8/23/21 | 18.27 | 79.29 | 2 | F | HET | 3210 |
| 19242 | 8/23/21 | 18.27 | 79.29 | 3 | F | HET | 3210 |
| 19243 | 8/23/21 | 18.27 | 79.29 | 5 | F | HET | 3210 |
| 19243 | 8/23/21 | 18.27 | 79.29 | 6 | F | HET | 3210 |
| 19243 | 8/23/21 | 18.27 | 79.29 | 7 | F | HET | 3210 |
| 19300 | 8/23/21 | 18.27 | 79.29 | 1 | M | HET | 3210 |
| 19300 | 8/23/21 | 18.27 | 79.29 | 2 | M | HET | 3210 |
|  |  |  |  |  |  |  |  |
