# Supplementary material for: A murine model lacking Lyst recapitulates Chediak-Higashi syndrome with an earlier-onset neurodegenerative phenotype
Source: Commun Biol. 2025 Jul 18;8:1064. doi: 10.1038/s42003-025-08482-1 (PMC12274407; doi:10.1038/s42003-025-08482-1)
Supplement: Supplementary file 7 — Supplementary Data 5 [file 42003_2025_8482_MOESM7_ESM.zip › B6 mice data/B6 HET Aging Study/B6 Aging Study_Transfer_5.8.23.docx]

| 19260 | 11/4/21 | 17.30 | 75.00 | 1 | F | HOM | 3239 |
| --- | --- | --- | --- | --- | --- | --- | --- |
| 19327 | 12/26/21 | 15.57 | 67.57 | 1 | M | HOM | 3239 |
| 19327 | 12/26/21 | 15.57 | 67.57 | 2 | M | HOM | 3239 |
| 19327 | 12/26/21 | 15.57 | 67.57 | 3 | M | HOM | 3239 |
| 19277 | 12/26/21 | 15.57 | 67.57 | 1 | F | HOM | 3239 |
| 19277 | 12/26/21 | 15.57 | 67.57 | 2 | F | HOM | 3239 |
| 19277 | 12/26/21 | 15.57 | 67.57 | 3 | F | HOM | 3239 |
| 19270 | 12/7/21 | 16.20 | 70.29 | 6 | F | HOM | 3253 |
|  |  |  |  |  |  |  |  |
| 19259 | 11/3/21 | 17.33 | 75.14 | 3 | F | WT | 3248 |
| 19259 | 11/3/21 | 17.33 | 75.14 | 4 | F | WT | 3248 |
| 19259 | 11/3/21 | 17.33 | 75.14 | 5 | F | WT | 3248 |
| 19275 | 12/21/21 | 15.73 | 68.29 | 1 | F | WT | 19289 |
| 19275 | 12/21/21 | 15.73 | 68.29 | 2 | F | WT | 19289 |
| 19275 | 12/21/21 | 15.73 | 68.29 | 3 | F | WT | 19289 |
| 19275 | 12/21/21 | 15.73 | 68.29 | 4 | F | WT | 19289 |
