# Supplementary material for: A murine model lacking Lyst recapitulates Chediak-Higashi syndrome with an earlier-onset neurodegenerative phenotype
Source: Commun Biol. 2025 Jul 18;8:1064. doi: 10.1038/s42003-025-08482-1 (PMC12274407; doi:10.1038/s42003-025-08482-1)
Supplement: Supplementary file 7 — Supplementary Data 5 [file 42003_2025_8482_MOESM7_ESM.zip › B6 mice data/B6 HET Aging Study/MS2302160-PathPrelim.pdf]

Pathology Preliminary Report

Division of Veterinary Resources, Office of Research Services, National Institutes of Health

Accession Number: MS2302160

NE / D

Date Submitted: 03/07/2023 **Date received by DVR: 03/08/2023** **Number of animals:** 3

Species: MOUSE **Source of animal(s):** **Date animal(s) received from source:**

Entry #	Animal ID	Sex	Strain	Age	Campus	Building	Room	Cage	Weight	Status	Date & Time of Death	Euthanasia Agent
1	EP#3	F	Hom X HOM	1 YR	Bethesda	49	3D13B	19269/1 A		L		
2	EP#2	M	Hom X HOM	1 YR	Bethesda	49	3D13B	17496/1 A		L		
3	DOB:10/22/21	M	Hom X HOM	1 YR	Bethesda	49	3D13B	19312/1 A		L		

Hazards: Infectious agent: **No** Radionuclide: **No** Chemical agent: **No** **Import Number:**

Remarks / History / Procedures:

3 single housed >1 yr old mice written up 3/7/23/am. for: CC# 19269-slight hunching, dehydration and tender abdomen, with minor rectal prolapse. Prolapse treated with panalog. CC# 17496-slight dehydration, enlarged bladder, OS slightly swollen with discharge, and slight increased RR. Bladder was expressed and dark yellow urine observed.3 X AB opthalmic ointment applied to eye. CC#19312- Slight hunching, abnormal breathing, lethargy, dehydration, distended abdomen with enlarged organs, (bladder distended). Urine was able to be expressed. Please perform full necropsy and check brain, spinal cord, please do pathology analysis for liver and spleen. Please use CAN# 8329269 if needed. *** Dr. Maclarty is now the APD. for NHGRI.

Preliminary Diagnosis:

A Syndrome, lymphoma,
B Harderian gland, hyperplasia, rule out adenoma, left
C Kidney, hydronephrosis, severe, unilateral, right
C Lung, possible eosinophilic macrophage pneumonia, right middle lobe

Preliminary Diagnosis Summary:

Three adult agouti mice are submitted live for evaluation. They are identified as a female mouse from Cage 19269, designated as A; a male mouse from Cage 17496, designated as B; and a male mouse from Cage 19312, designated as C. The findings are as follows:

Mouse A: The mouse is relatively well hydrated and well-muscled and contains a small amount of body fat. The left submandibular lymph node is moderately enlarged. The mesenteric lymph node is moderate to severely enlarged uniformly. The spleen is moderately enlarged uniformly. A mass is present in the region of the thymus measuring 0.7 x 0.5 x 0.2 cm on the left side. The heart, lungs, liver, kidneys, GI tract, uterus and ovaries and brain appear grossly normal. There is minimal eversion of the rectum in the 5 to 7 o'clock position of the anus. A sample of colonic content is obtained for PCR for Helicobacter. The stomach contains a moderate amount of ingesta. Small intestines, cecum and colon appear normal. Tissues are collected for histopathology.

Mouse B: The mouse has a mildly protruding left eye. The eyes appear grossly normal. Examination reveals the left Harderian gland to be mildly enlarged. Hydration is fair. The heart, lungs, liver, kidneys, spleen, GI tract, testes and brain appear grossly normal. Select tissues are obtained for histopathology.

Mouse C: Hydration is good. The mouse is relatively well-muscled and contains adequate body fat. The right kidney is severely enlarged and distended with urine. It is reddish-brown and measures 3.0 x 2.5 x 2.0 cm. The left kidney appears normal. The heart, liver, spleen, GI tract, testes and brain appear grossly normal. A small amount of ingesta is present within the stomach and formed content is present in the colon. The urinary bladder is mild to moderately distended with urine. A 2 x 2 mm region of the right middle lung lobe appears gray and slightly consolidated, possibly due to eosinophilic macrophage pneumonia. The remainder of the lung appears normal. Select tissues are obtained for histopathology.

Diagnostician: Eckhaus, Michael
