# Supplementary figures and images for: A murine model lacking Lyst recapitulates Chediak-Higashi syndrome with an earlier-onset neurodegenerative phenotype
Source: Commun Biol. 2025 Jul 18;8:1064. doi: 10.1038/s42003-025-08482-1 (PMC12274407; doi:10.1038/s42003-025-08482-1)

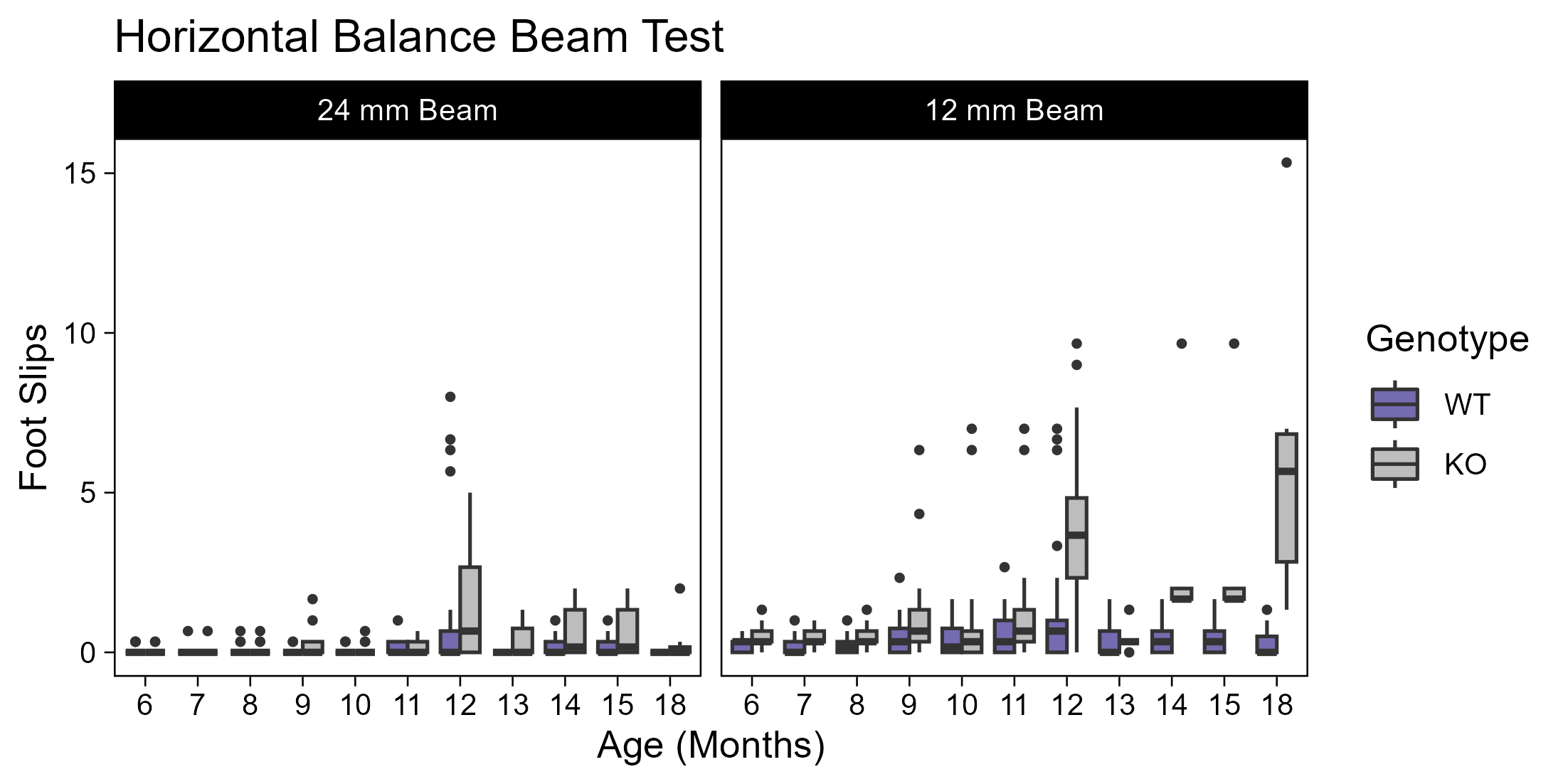

Supplement: Supplementary file 7 — Supplementary Data 5 [file 42003_2025_8482_MOESM7_ESM.zip › B6 mice data/B6 Behavior Study/Beam_Foot_Slips_Data_Plot_Lyst-KO.png]

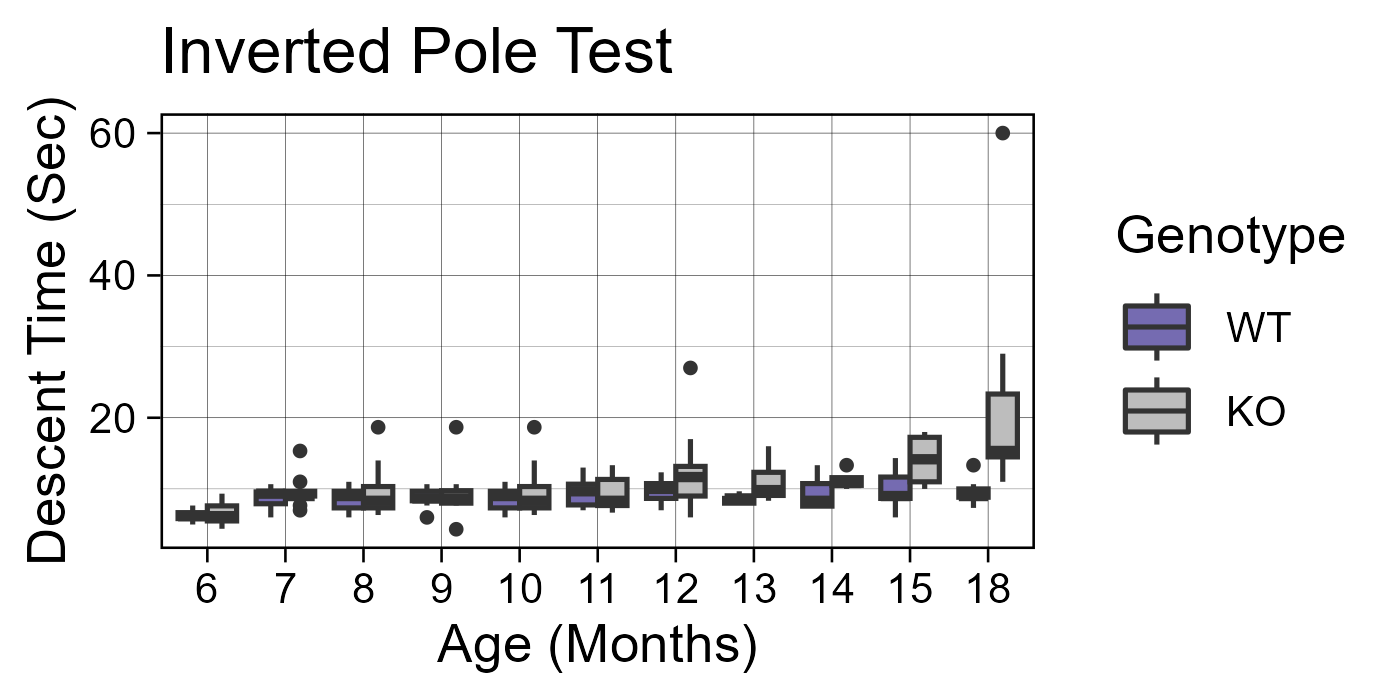

Supplement: Supplementary file 7 — Supplementary Data 5 [file 42003_2025_8482_MOESM7_ESM.zip › B6 mice data/B6 Behavior Study/Pole_Data_Descent_Plot_Lyst-KO.png]

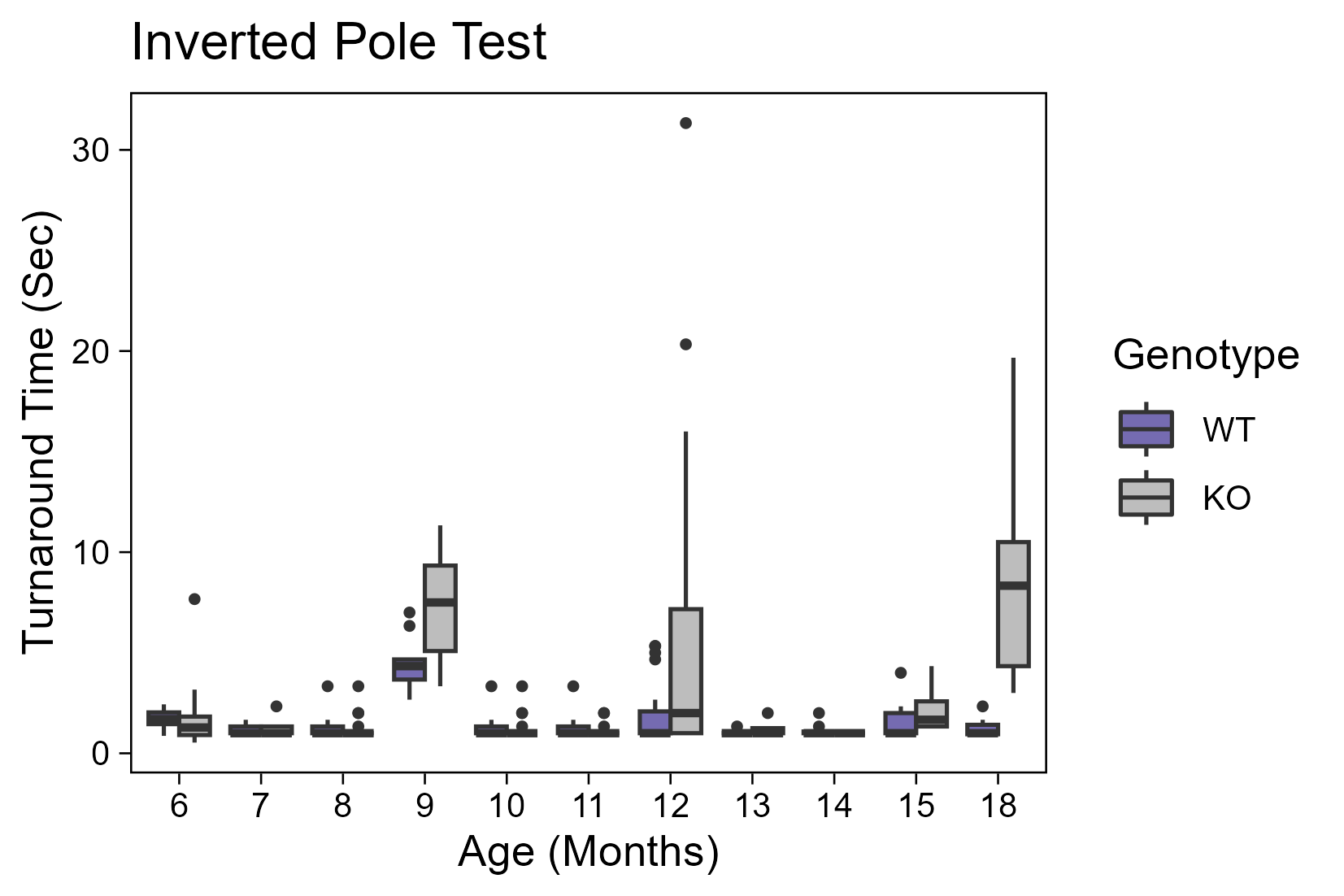

Supplement: Supplementary file 7 — Supplementary Data 5 [file 42003_2025_8482_MOESM7_ESM.zip › B6 mice data/B6 Behavior Study/Pole_Data_TAT_Plot_Lyst-KO.png]

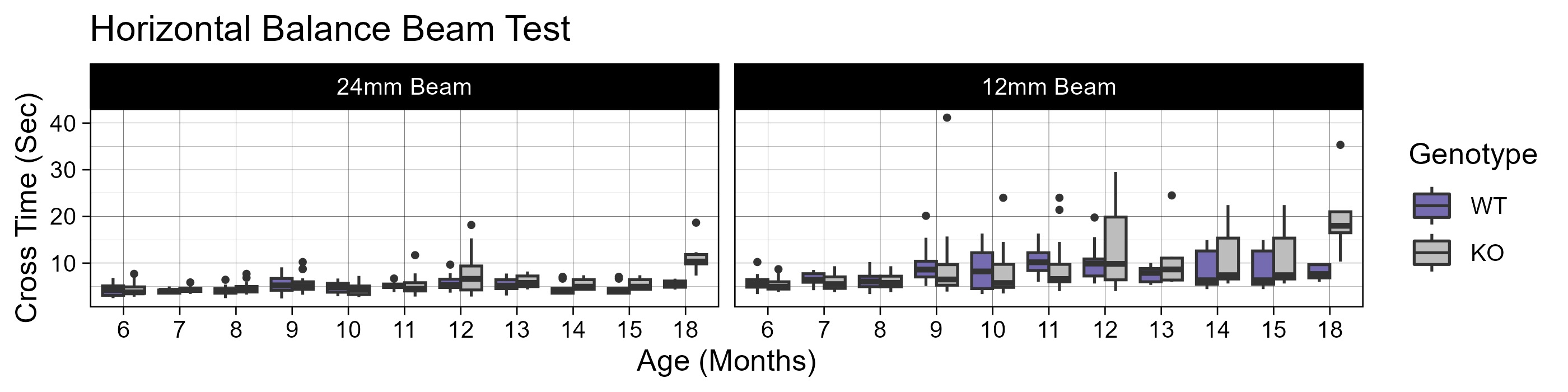

Supplement: Supplementary file 7 — Supplementary Data 5 [file 42003_2025_8482_MOESM7_ESM.zip › B6 mice data/B6 Behavior Study/Beam_Cross_Time_Data_Plot_Lyst-KO.png]

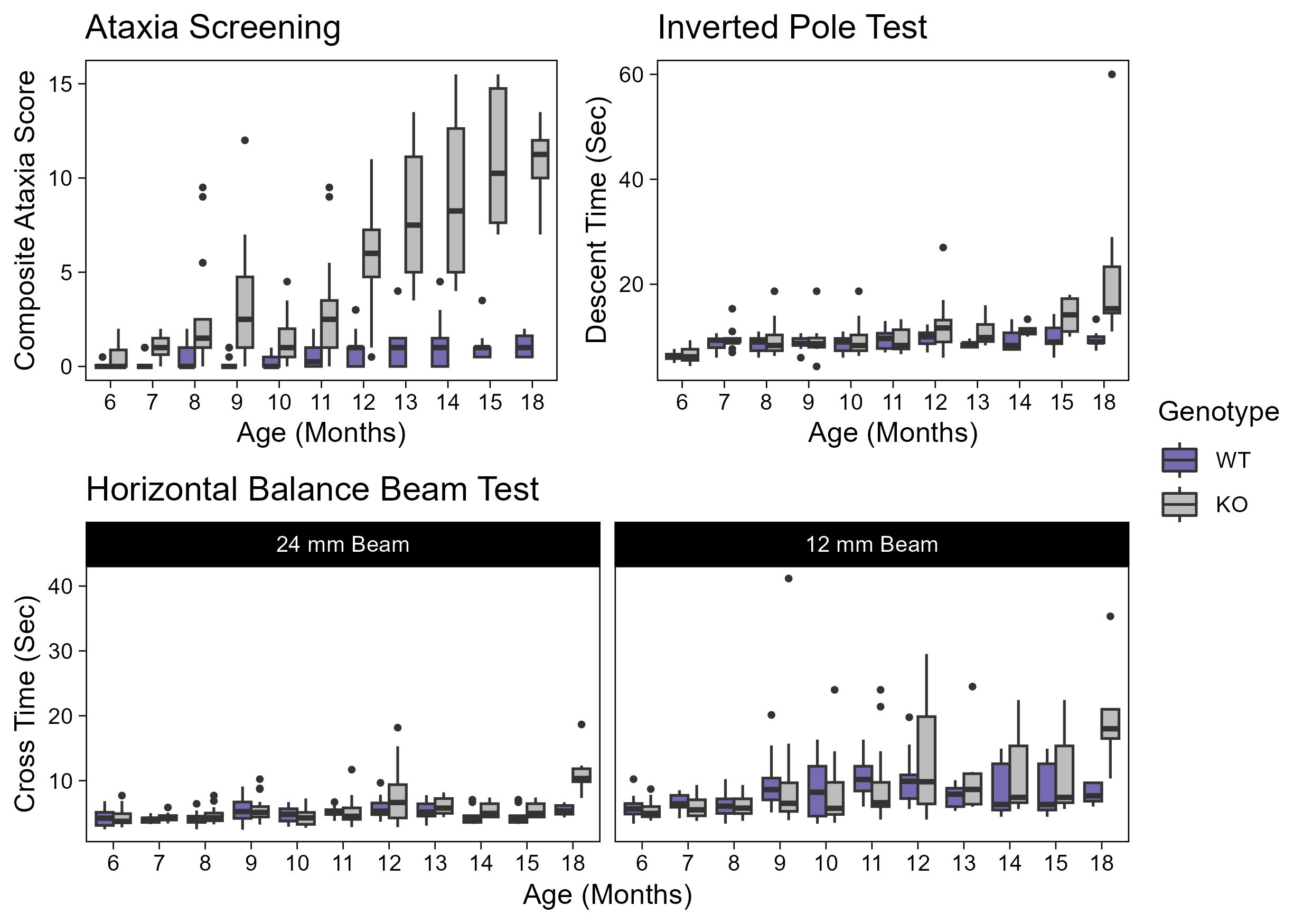

Supplement: Supplementary file 7 — Supplementary Data 5 [file 42003_2025_8482_MOESM7_ESM.zip › B6 mice data/B6 Behavior Study/ComboPlot_AtaxiaScreen-PoleDescent-BeamTime_Lyst-KO.png]

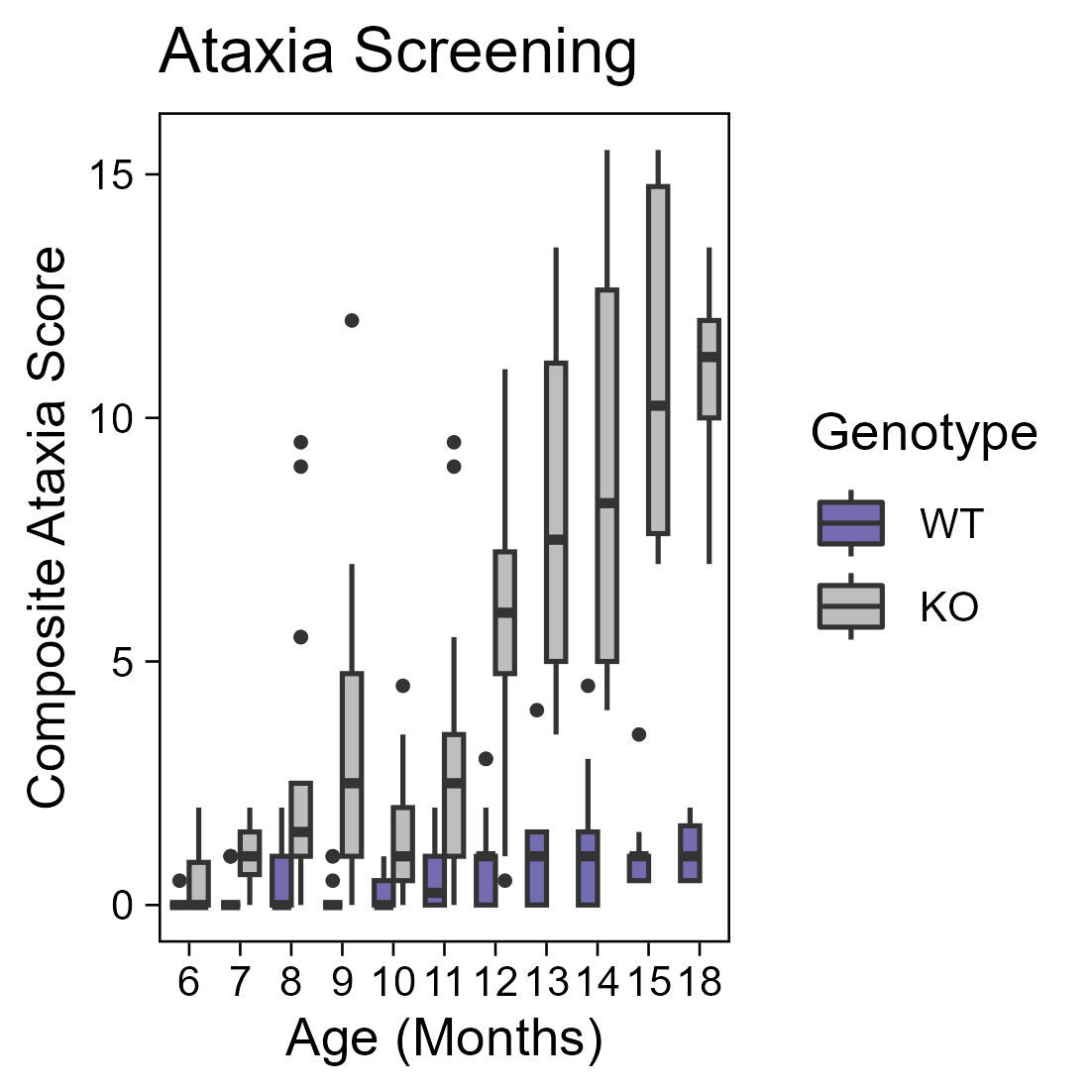

Supplement: Supplementary file 7 — Supplementary Data 5 [file 42003_2025_8482_MOESM7_ESM.zip › B6 mice data/B6 Behavior Study/Ataxia_Score_Plot_Lyst-KO.png]

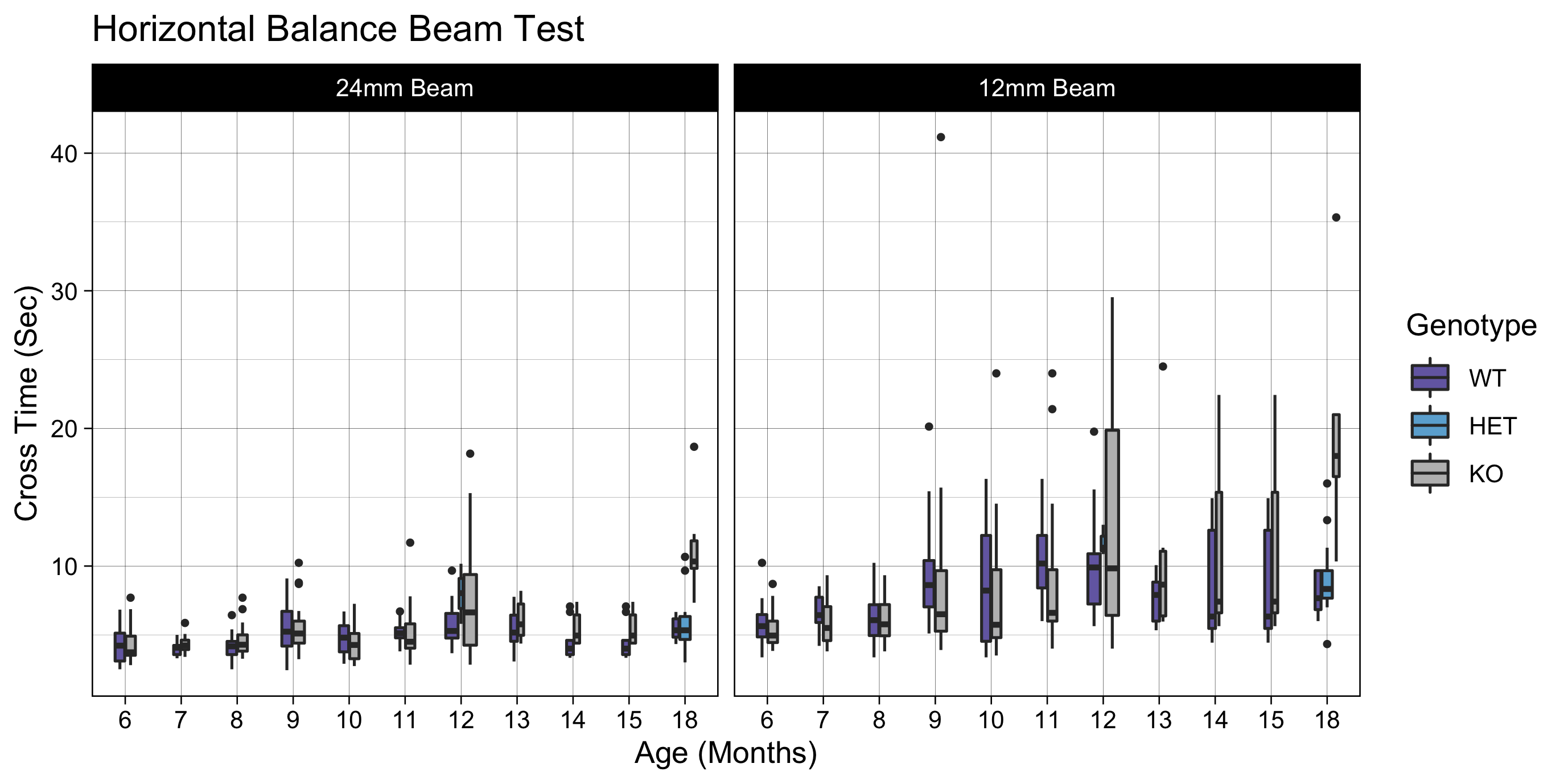

Supplement: Supplementary file 7 — Supplementary Data 5 [file 42003_2025_8482_MOESM7_ESM.zip › B6 mice data/B6 HET Aging Study/image002.png]

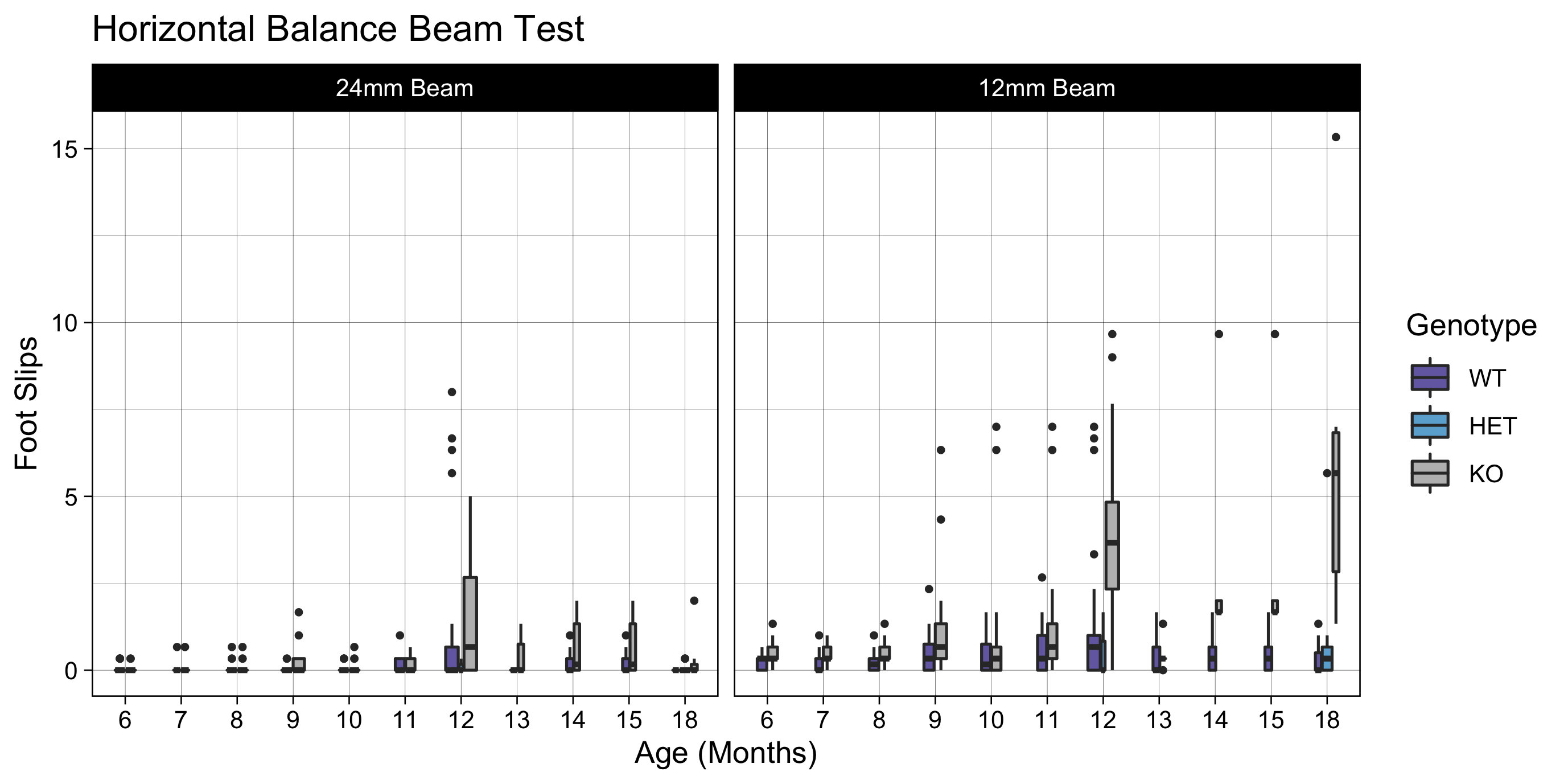

Supplement: Supplementary file 7 — Supplementary Data 5 [file 42003_2025_8482_MOESM7_ESM.zip › B6 mice data/B6 HET Aging Study/image001.png]

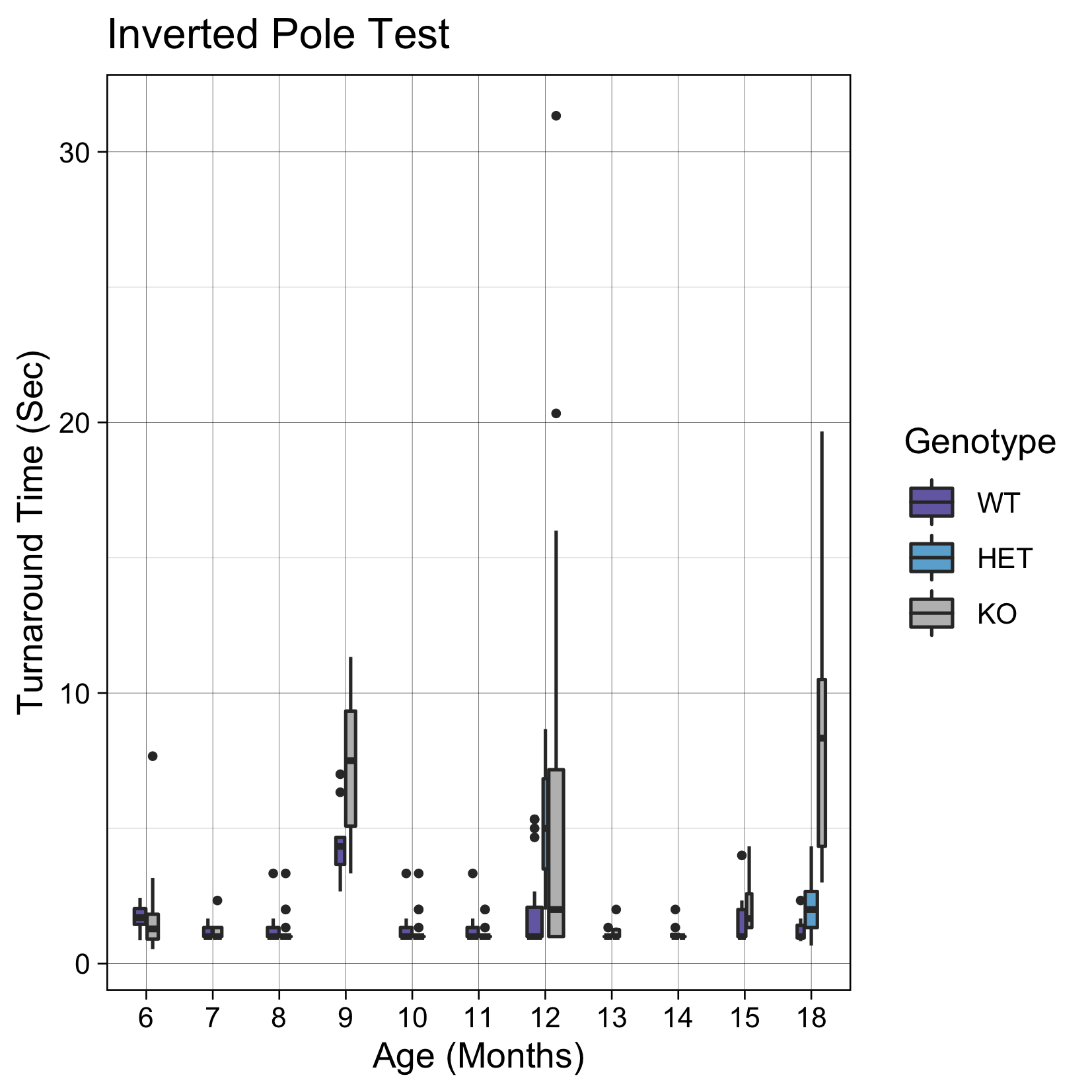

Supplement: Supplementary file 7 — Supplementary Data 5 [file 42003_2025_8482_MOESM7_ESM.zip › B6 mice data/B6 HET Aging Study/image004.png]

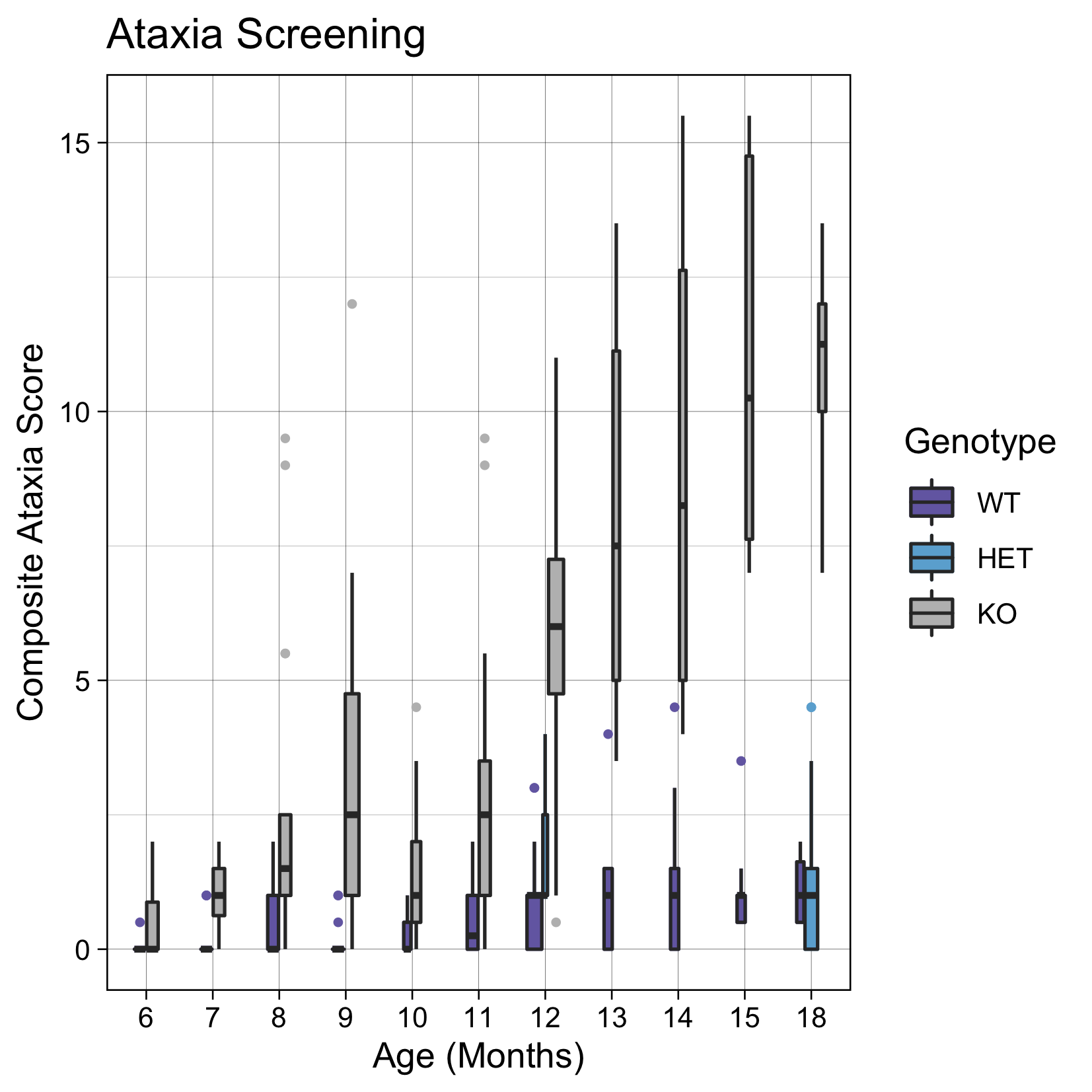

Supplement: Supplementary file 7 — Supplementary Data 5 [file 42003_2025_8482_MOESM7_ESM.zip › B6 mice data/B6 HET Aging Study/image005.png]

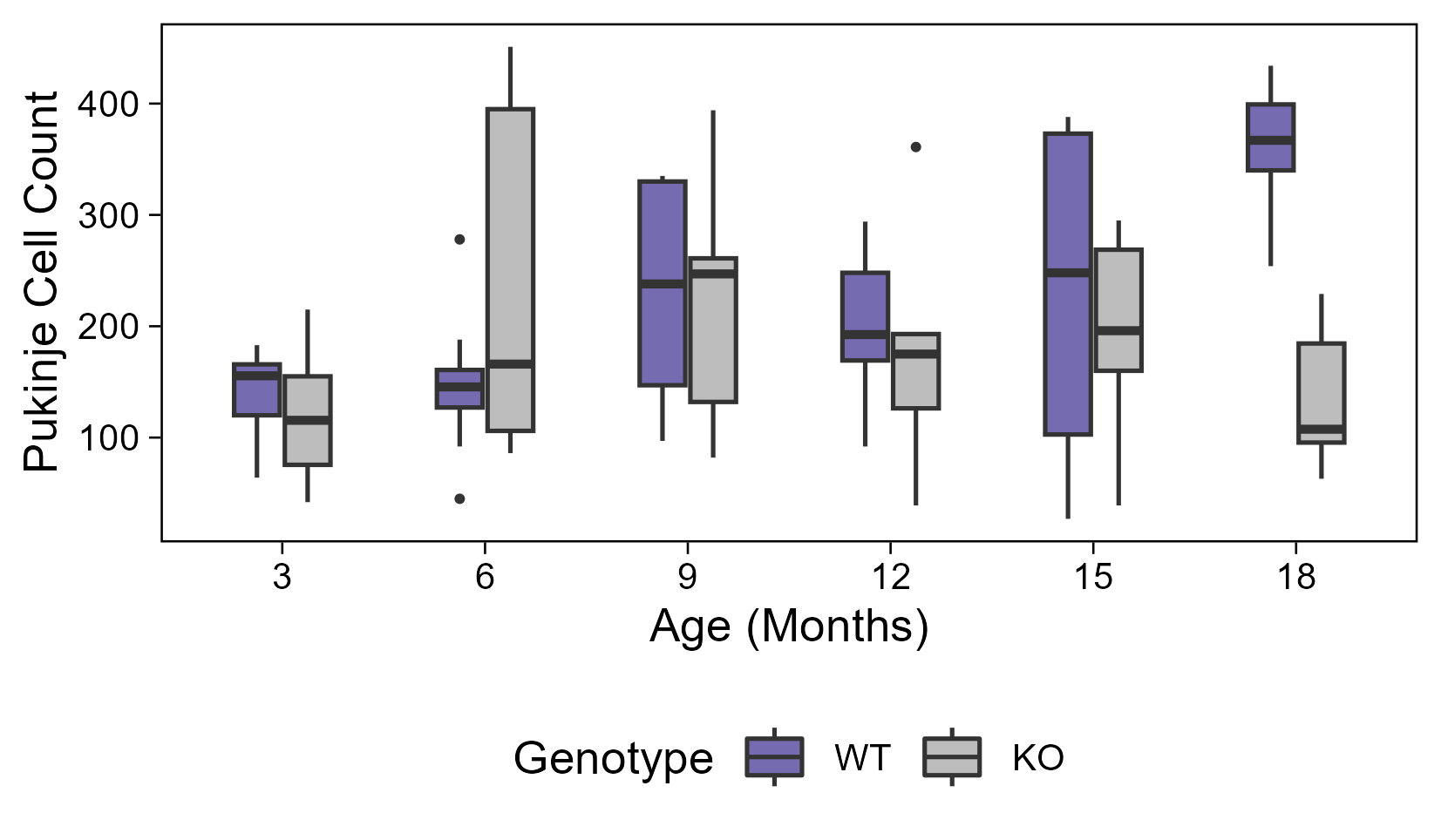

Supplement: Supplementary file 7 — Supplementary Data 5 [file 42003_2025_8482_MOESM7_ESM.zip › B6 mice data/Purkinje Cell Counting/Purkinje_Cell_Counts_Plot_Lyst-KO.png]

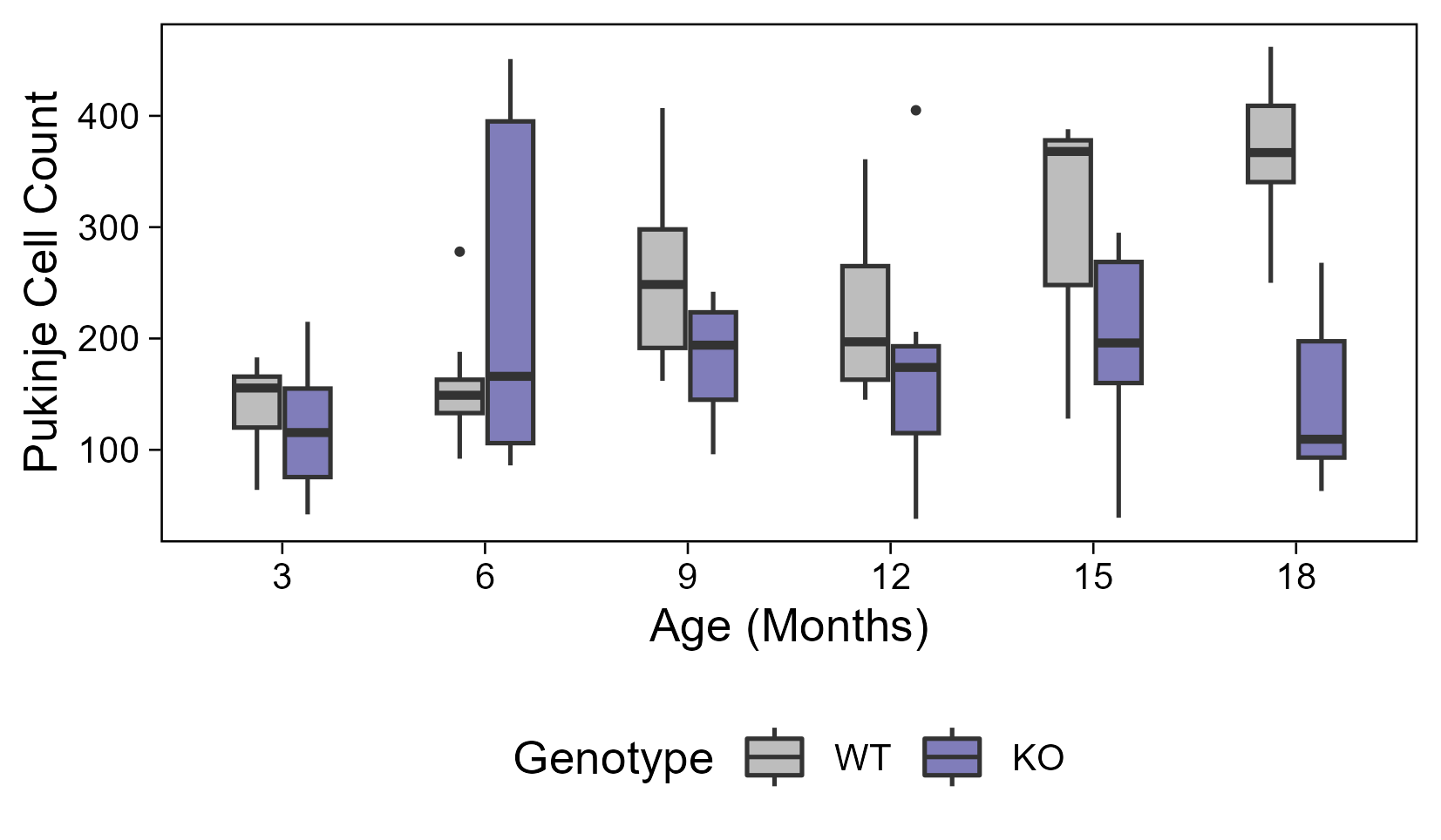

Supplement: Supplementary file 7 — Supplementary Data 5 [file 42003_2025_8482_MOESM7_ESM.zip › B6 mice data/Purkinje Cell Counting/Purkinje_Cell_Counts_Plot_Lyst-KO_reverse coloring.png]

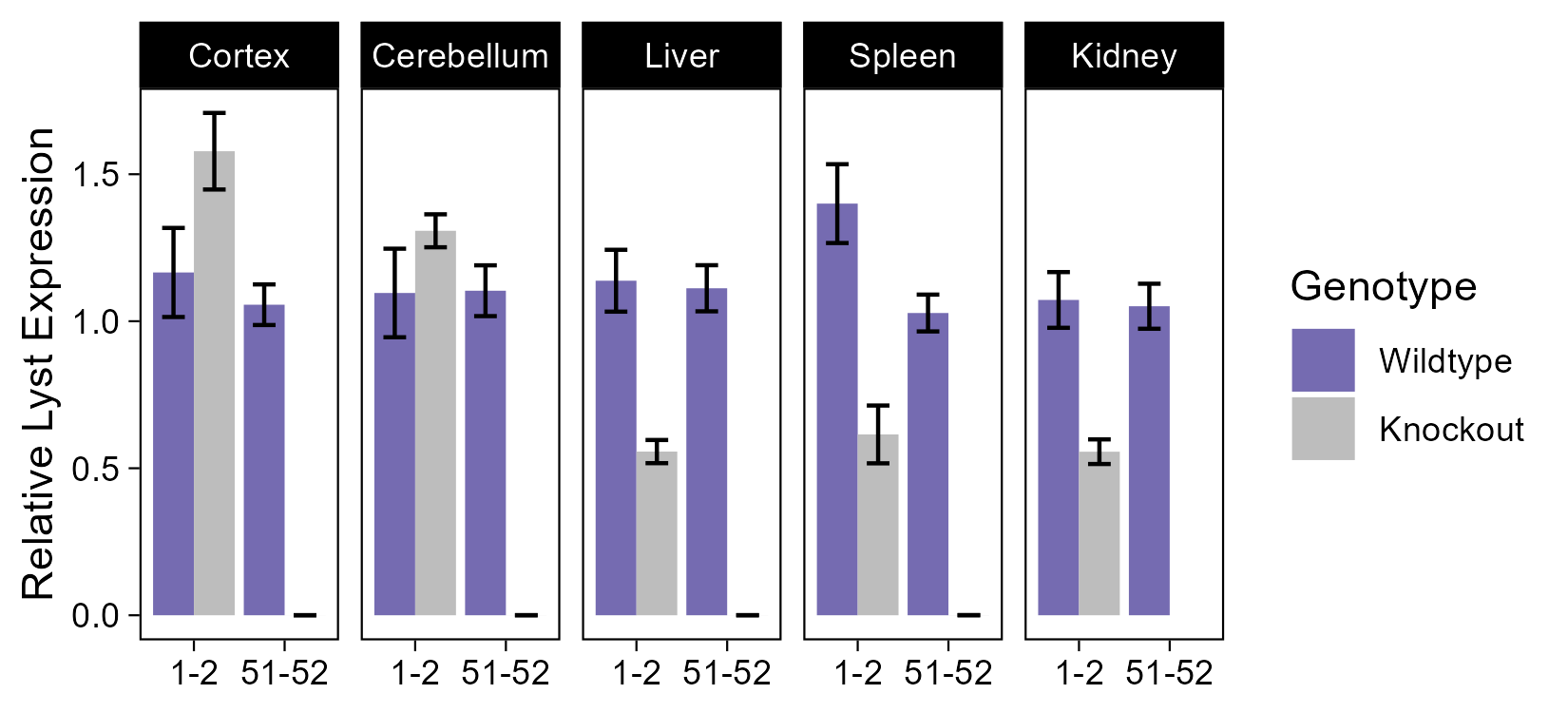

Supplement: Supplementary file 7 — Supplementary Data 5 [file 42003_2025_8482_MOESM7_ESM.zip › B6 mice data/qPCR/Tissue-wise_lyst_Expression_KO_Mice.png]

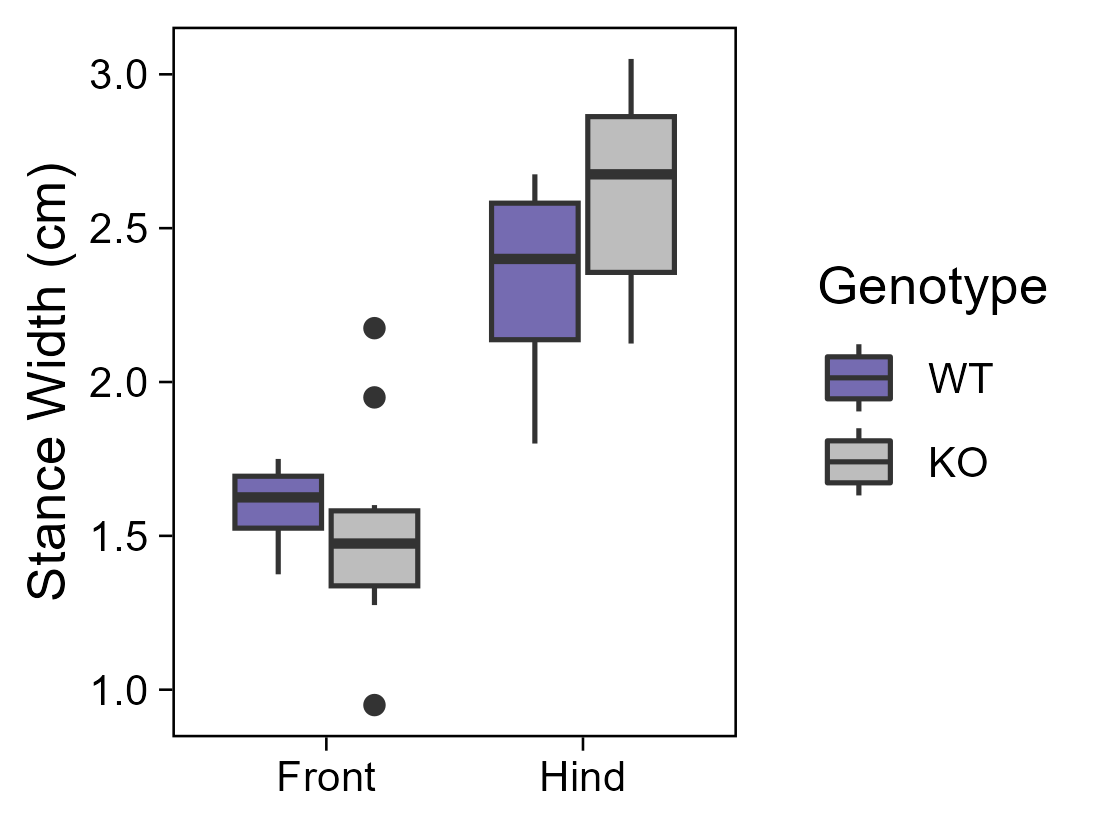

Supplement: Supplementary file 7 — Supplementary Data 5 [file 42003_2025_8482_MOESM7_ESM.zip › B6 mice data/Paw Analysis Averages/Old_Mice_Stance_Widths_Plot_Average.png]

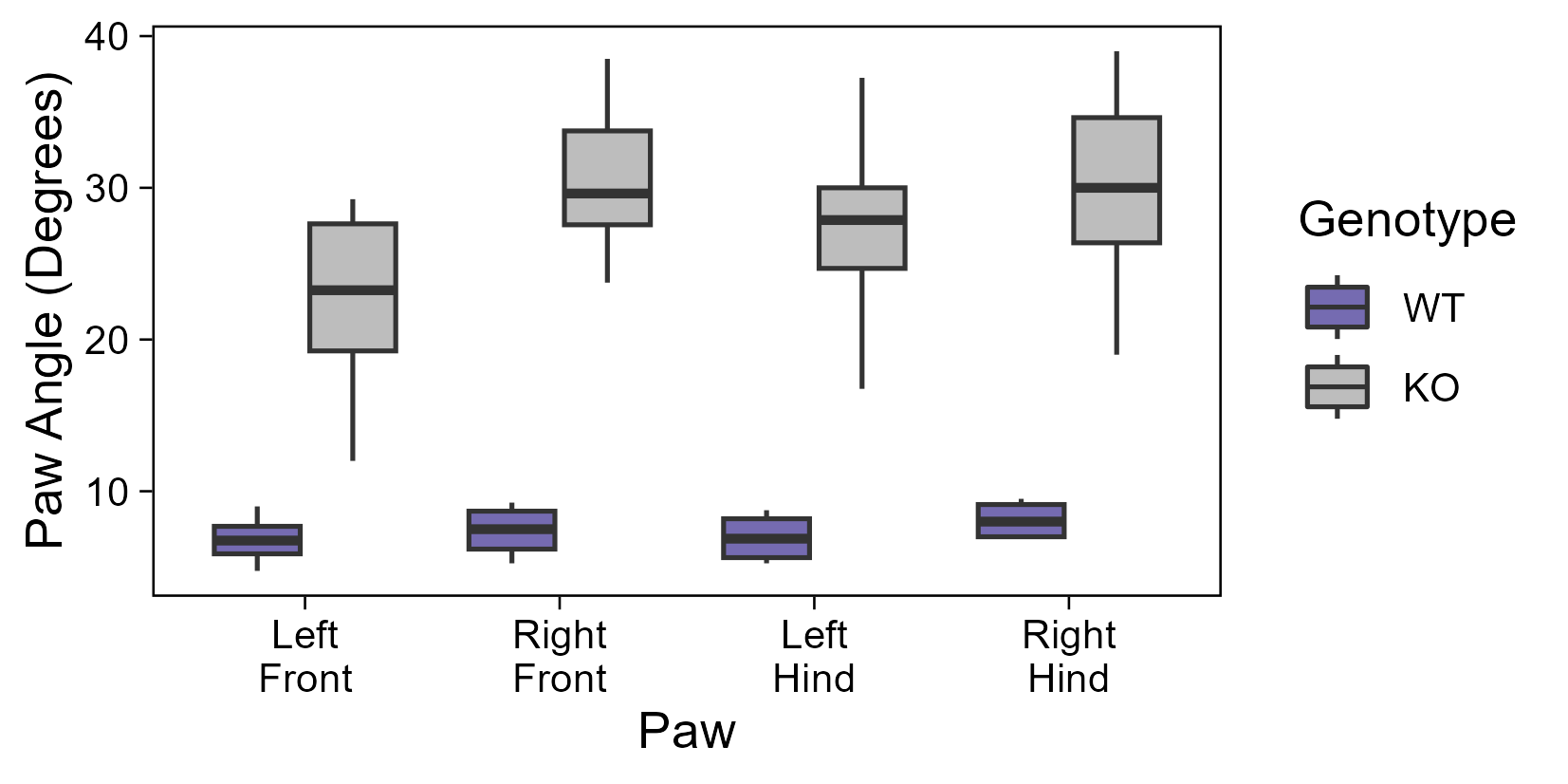

Supplement: Supplementary file 7 — Supplementary Data 5 [file 42003_2025_8482_MOESM7_ESM.zip › B6 mice data/Paw Analysis Averages/Old_Mice_Paw_Angles_Plot_Average.png]

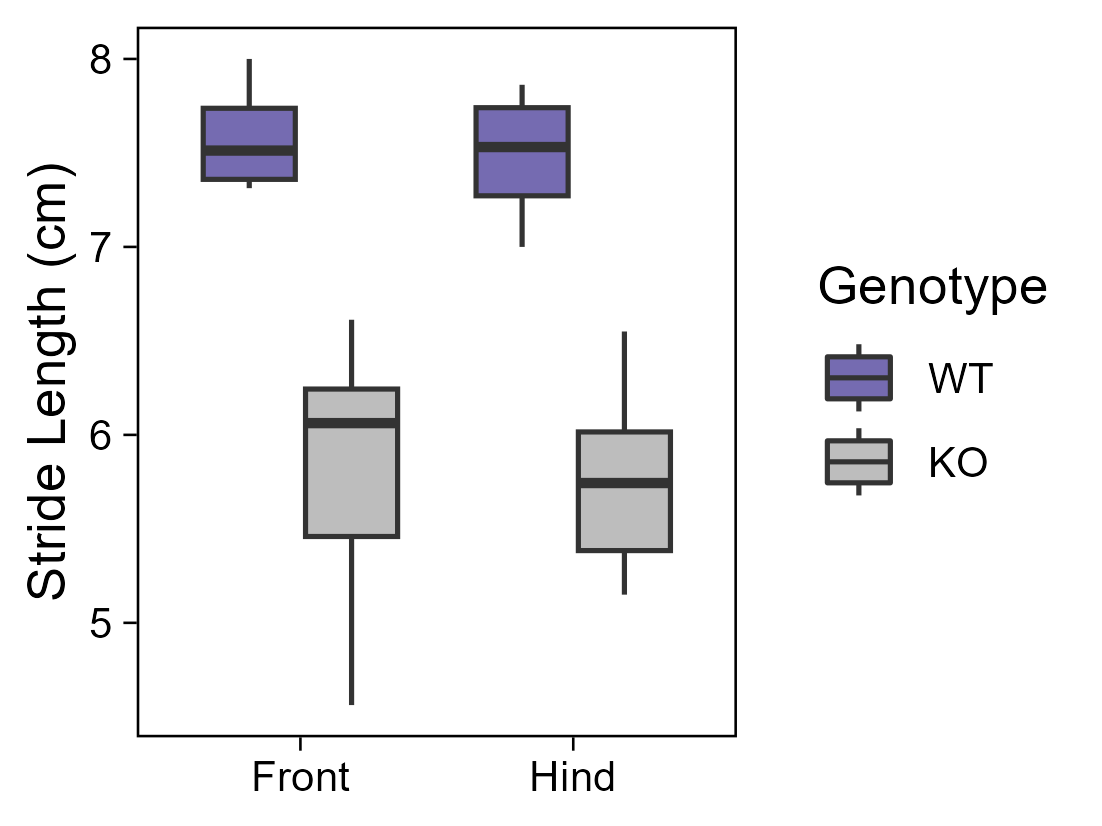

Supplement: Supplementary file 7 — Supplementary Data 5 [file 42003_2025_8482_MOESM7_ESM.zip › B6 mice data/Paw Analysis Averages/Old_Mice_Stride_Lengths_Plot_Average.png]

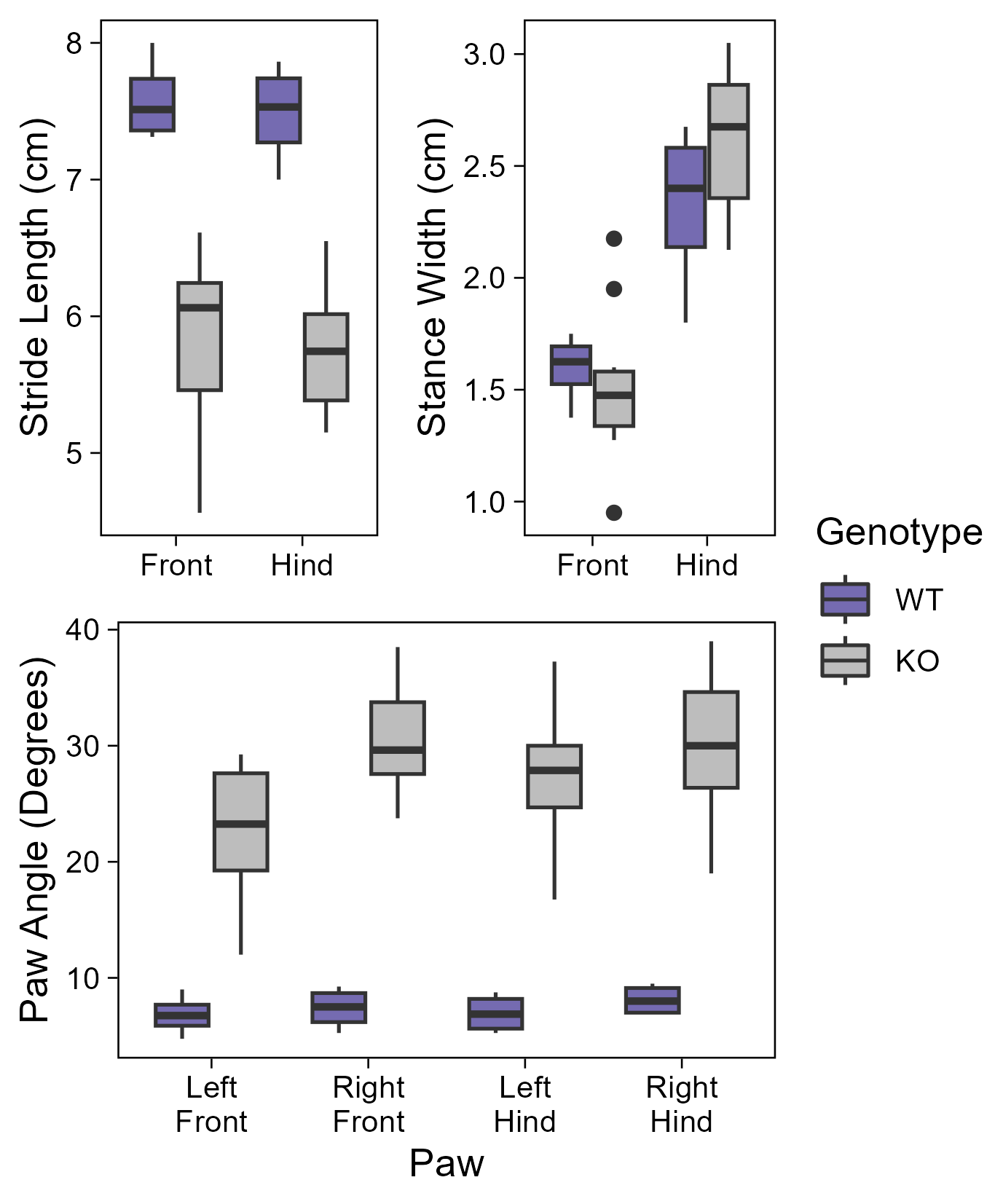

Supplement: Supplementary file 7 — Supplementary Data 5 [file 42003_2025_8482_MOESM7_ESM.zip › B6 mice data/Paw Analysis Averages/ComboPlot_StrideLength-StanceWidtht-PawAngle_Lyst-KO_Average.png]

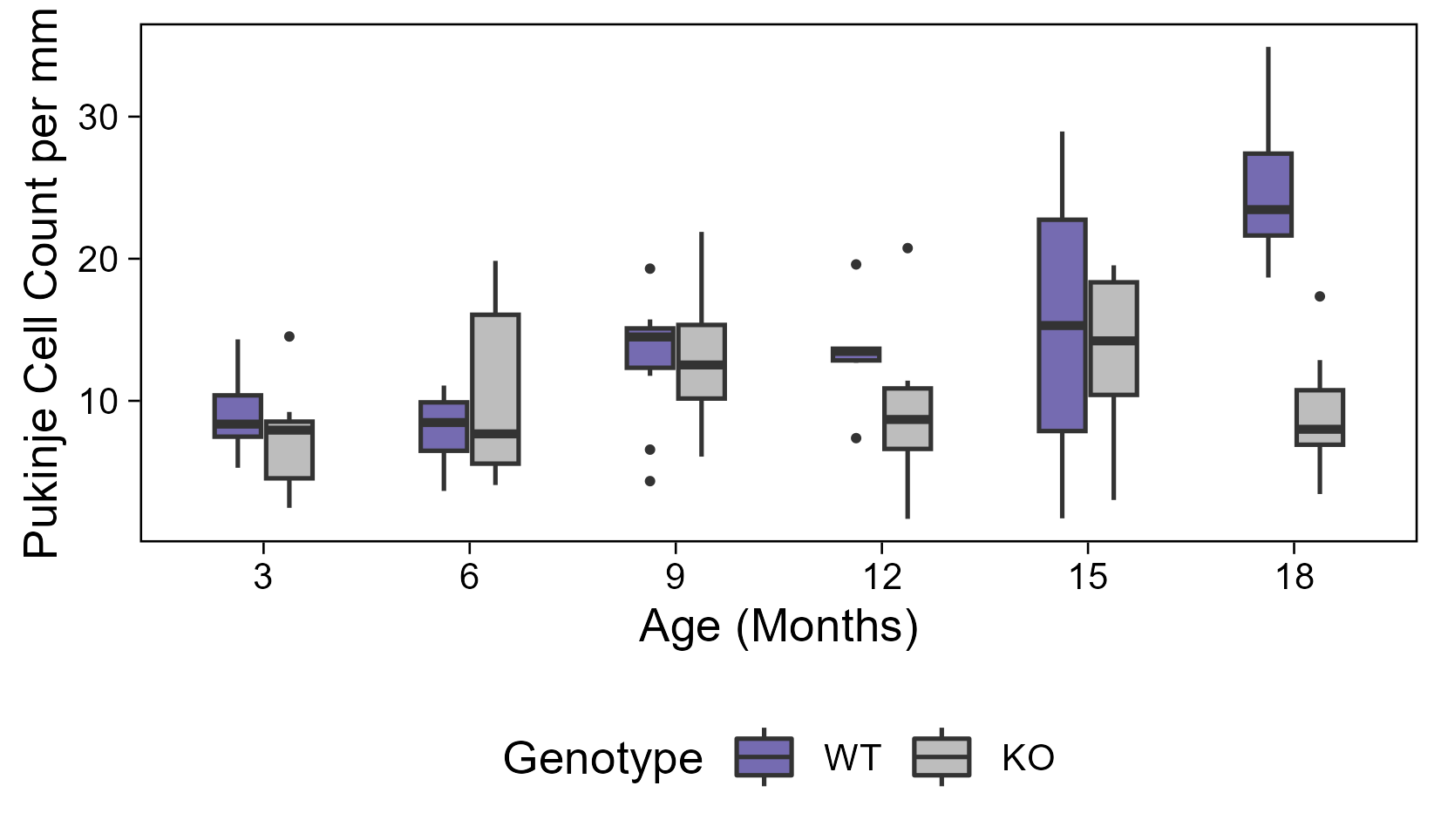

Supplement: Supplementary file 7 — Supplementary Data 5 [file 42003_2025_8482_MOESM7_ESM.zip › B6 mice data/Purkinje Cell Counting/Purkinje Density/Purkinje_Cell_Density_Plot_Lyst-KO.png]

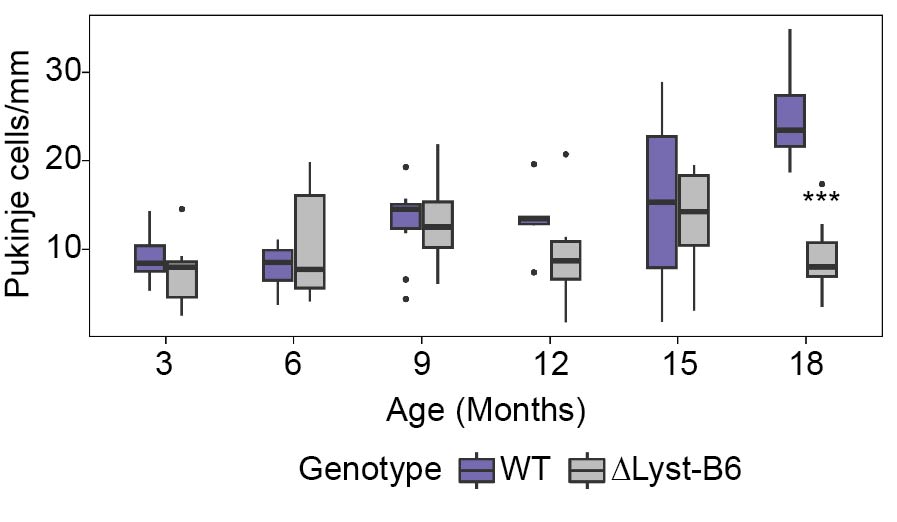

Supplement: Supplementary file 7 — Supplementary Data 5 [file 42003_2025_8482_MOESM7_ESM.zip › B6 mice data/Purkinje Cell Counting/Purkinje Density/Purkinje cell per mm.jpg]
