# Supplementary material for: A murine model lacking Lyst recapitulates Chediak-Higashi syndrome with an earlier-onset neurodegenerative phenotype
Source: Commun Biol. 2025 Jul 18;8:1064. doi: 10.1038/s42003-025-08482-1 (PMC12274407; doi:10.1038/s42003-025-08482-1)
Supplement: Supplementary file 8 — Description of Additional Supplementary Files [file 42003_2025_8482_MOESM8_ESM.pdf]

Description of Additional Supplementary Files

Supplementary Data 1. Deleted allele sequence

Supplementary Data 2. Differentially expressed genes from RNA-seq analysis of the forebrain and cerebellum in 3- and 18-month WT and ΔLYST-B6 mice.

Supplemental Data 3. Gene Ontology (GO) Biological Process (BP) terms that are enriched from RNA-seq analysis of the forebrain and cerebellum in 3- and 18-month-old WT and ΔLYST-B6 mice.

Supplemental Data 4a. Differentially expressed lipid classes.

Supplemental Data 4b. Differentially expressed individual lipids.

Supplemental Data 5. Source files for figures.
