# Supplementary material for: A murine model lacking Lyst recapitulates Chediak-Higashi syndrome with an earlier-onset neurodegenerative phenotype
Source: Commun Biol. 2025 Jul 18;8:1064. doi: 10.1038/s42003-025-08482-1 (PMC12274407; doi:10.1038/s42003-025-08482-1)
Supplement: Supplementary file 9 — Reporting summary [file 42003_2025_8482_MOESM9_ESM.pdf]

nature portfolio

Corresponding author(s): May Christine Malicdan

Last updated by author(s): Jan 8, 2025

Reporting Summary

Nature Portfolio wishes to improve the reproducibility of the work that we publish. This form provides structure for consistency and transparency in reporting. For further information on Nature Portfolio policies, see our Editorial Policies and the Editorial Policy Checklist.

Statistics

For all statistical analyses, confirm that the following items are present in the figure legend, table legend, main text, or Methods section.

n/a	Confirmed	
☐	☒	The exact sample size (*n*) for each experimental group/condition, given as a discrete number and unit of measurement
☐	☒	A statement on whether measurements were taken from distinct samples or whether the same sample was measured repeatedly
☐	☒	The statistical test(s) used AND whether they are one- or two-sided<br>*Only common tests should be described solely by name; describe more complex techniques in the Methods section.*
☐	☒	A description of all covariates tested
☐	☒	A description of any assumptions or corrections, such as tests of normality and adjustment for multiple comparisons
☐	☒	A full description of the statistical parameters including central tendency (e.g. means) or other basic estimates (e.g. regression coefficient) AND variation (e.g. standard deviation) or associated estimates of uncertainty (e.g. confidence intervals)
☐	☒	For null hypothesis testing, the test statistic (e.g. F, t, r) with confidence intervals, effect sizes, degrees of freedom and P value noted<br>*Give P values as exact values whenever suitable.*
☒	☐	For Bayesian analysis, information on the choice of priors and Markov chain Monte Carlo settings
☒	☐	For hierarchical and complex designs, identification of the appropriate level for tests and full reporting of outcomes
☐	☒	Estimates of effect sizes (e.g. Cohen's *d*, Pearson's *r*), indicating how they were calculated

Our web collection on statistics for biologists contains articles on many of the points above.

Software and code

Policy information about availability of computer code

Data collection	N/A
Data analysis	All data analysis and visualization was performed in RStudio. RNA library preparation and standard bulk RNA-sequencing was performed on the Illumina NovaSeq platform. Differential expression was determined using the results() function in the DESeq2 R package. All differentially-expressed genes (adjusted P ≤ 0.05) were then tested for functional enrichment using the WebGestaltR R package. Metascape, an online gene and pathway annotation tool, was also used for comprehensive visualization. Code for Purkinje cell quantification is available at: https://github.com/mkhapp/Purkinje_Cell_Linear_Density/.

For manuscripts utilizing custom algorithms or software that are central to the research but not yet described in published literature, software must be made available to editors and reviewers. We strongly encourage code deposition in a community repository (e.g. GitHub). See the Nature Portfolio guidelines for submitting code & software for further information.

Data

Policy information about availability of data

All manuscripts must include a data availability statement. This statement should provide the following information, where applicable:

- Accession codes, unique identifiers, or web links for publicly available datasets
- A description of any restrictions on data availability
- For clinical datasets or third party data, please ensure that the statement adheres to our policy

All data and accompanying information for the transcriptomic and lipidomic datasets used in this study is publicly available and deposited in Gene Expression Omnibus (GEO accession number GSE266142).

Research involving human participants, their data, or biological material

Policy information about studies with human participants or human data. See also policy information about sex, gender (identity/presentation), and sexual orientation and race, ethnicity and racism.

Reporting on sex and gender	N/A
Reporting on race, ethnicity, or other socially relevant groupings	N/A
Population characteristics	N/A
Recruitment	N/A
Ethics oversight	N/A

Note that full information on the approval of the study protocol must also be provided in the manuscript.

Field-specific reporting

Please select the one below that is the best fit for your research. If you are not sure, read the appropriate sections before making your selection.

☒ Life sciences ☐ Behavioural & social sciences ☐ Ecological, evolutionary & environmental sciences

For a reference copy of the document with all sections, see nature.com/documents/nr-reporting-summary-flat.pdf

Life sciences study design

All studies must disclose on these points even when the disclosure is negative.

Sample size	Sample size was determined by resource availability and feasibility of experiments. The sample size of animals for various experiments was deemed sufficient to demonstrate reproducibility of acquired results and to provide sufficient data for calculated power while also considering ethical use and treatment of experimental animals.
Data exclusions	Histological images of tissue that were severely damaged in preparation of tissues and slides were excluded from quantification (nerve fibril/ Purkinje cells) data analysis. This exclusion was to reduce the influence of artifact and tissue preparation in statistical analysis. During behavioral testing using the balance beam, animals who scooted across the beam were excluded from analysis due to the inappropriate crossing of the beam not accepted by the methodology.
Replication	The qPCR and Western Blot experiments were repeated with consistent results across the tissue types of 3-6 animals per sex/strain. Experiments, including behavioral assays and histological analyses, were repeated in separate trials with consistent results across cohorts of 5-10 animals per sex/strain/age group.
Randomization	Experimental groups were determined by animal genotype; however, covariates were controlled by using inbred transgenic animals. No official randomization was done.
Blinding	Nerve fibril quantification and Purkinje cell quantification was performed by a blinded investigator. Histological imaging of the brain, retina, and blood smear was performed by a blinded investigator. Investigator blinding was not possible for behavioral testing due to the visually distinct phenotypic coat color of the mutant mice.

Reporting for specific materials, systems and methods

We require information from authors about some types of materials, experimental systems and methods used in many studies. Here, indicate whether each material, system or method listed is relevant to your study. If you are not sure if a list item applies to your research, read the appropriate section before selecting a response.

Materials & experimental systems

n/a	Involved in the study	
☐	☒	Antibodies
☐	☒	Eukaryotic cell lines
☒	☐	Palaeontology and archaeology
☐	☒	Animals and other organisms
☒	☐	Clinical data
☒	☐	Dual use research of concern
☒	☐	Plants

Methods

n/a	Involved in the study	
☒	☐	ChIP-seq
☒	☐	Flow cytometry
☒	☐	MRI-based neuroimaging

Antibodies

Antibodies used

Primary Antibodies:
Anti-LYST Rabbit Polyclonal, Atlas Antibodies, HPA055725; Anti-LYST Rabbit Polyclonal, Novus Biologicals, NBP2-56533; Anti-Vinculin mouse monoclonal, Sigma, SAB4200080, clone V284, purified; Anti-LAMP1, Developmental Studies Hybridoma Bank 1D4B; Anti-Calbindin D-28k Rabbit, Swant CB38; Anti-PCP4 Thermo Scientific, PA5-52209; Anti-GFP Chicken polyclonal, Invitrogen, PA1-10004.

Secondary Antibodies:
IRDYE 800CW-conjugated donkey anti-rabbit IgG, LI-COR Biosciences, 926-32213; IRDYE 680RD-conjugated donkey anti-mouse, LI-COR Biosciences, 92-68072; Alexa Fluor 488, Invitrogen, A21208; Alexa Fluor Phalloidin 555, Invitrogen, A34055; Alexa Fluor 633, Invitrogen, A11039; Alexa Fluor 555, Abcam, Ab150154; Alexa Fluor 488, Invitrogen, A21070

Validation

Validation of primary signal from primary antibodies was validated with using no primary antibody (or only secondary antibody). In addition, here are the details of the antibodies used.
Anti-LYST Atlas Antibodies, HPA055725; Affinity was purified using the PrEST-Antigen as affinity ligand and is validated in immunocytochemistry; RRID# AB_2682898; there are no previous publication using this antibody. Validation in this manuscript is the protein immunoblot of various wildtype and mutant animal tissues and mouse embryonic fibroblasts.

Anti-LYST Rabbit Polyclonal, Novus Biologicals, NBP2-56533 QC tested for reactivity to human LYST with predicted reactivity in mouse of 95%; recommended for immunocytochemistry /immunofluorescence; validated by immunostaining of human cell line HEK 293; no publications affiliated. Validation in this manuscript is the protein immunoblot of various wildtype and mutant animal tissues and mouse embryonic fibroblasts.

Anti-Vinculin mouse monoclonal, Sigma, SAB4200080, clone V284, purified; QC tested reactivity to mouse vinculin; recommended for immunocytochemistry/ western blot; published by the authors in Avalle L et al., Cell Death and Differentiation, 2019; UNSPSC Code: 12352203.

Anti-LAMP1, Developmental Studies Hybridoma Bank 1D4B; QC tested reactivity to mouse LAMP1; recommended for immunofluorescence, immunohistochemistry; cited in 717 publications and published by authors Toledano-Aragoza et al., Cell Death & Disease, 2024; Antibody Registry ID: AB_528127.

Anti-Calbindin D-28k Rabbit, Swant CB38; QC tested reactivity to mouse Albindin D-28k; published application in immunohistochemistry of paraffinized and frozen sections; cited in 44 publications and published by Soleilhavoup et al, Nature Communications, 2020.

Anti-PCP4 Thermo Scientific, PA5-52209; verified by relative expression, QC tested reactivity to mouse recombinant protein corresponding to Human PCP4; recommended for immunofluorescence/ immunohistochemistry; cited in 4 publications and published by authors Radzicki et al, Hippocampus, 2023; RRID# AB_2645298.

Anti-GFP, Invitrogen, PA1-10004; verified by relative expression, verified reactivity to mouse recombinant full length human GFAP isotype 1; recommended for immunohistochemistry, immunocytochemistry, western blot; cited in 40 publications and published by authors Sanchez-Petidier et al, Stem Cel Research & Therapy, 2022; RRID# AB_1074620.

Eukaryotic cell lines

Policy information about cell lines and Sex and Gender in Research

Cell line source(s)

Mouse Embryonic Fibroblasts were derived from wildtype inbred C57Bl/6J (Jackson laboratories, 000664) and inbred mutant □LYST-B6 (C57Bl/6J-Lystem1-40mal) embryos. Pregnant mice were euthanized at 15.5 days of gestation, embryos harvested from the uterus, euthanized, and tissues were minced into a slurry and plated in culture with complete Dulbecco's modified Eagle's medium supplemented by 15% fetal bovine serum, 100 IU/mL penicillin, and 100 ug/mL streptomycin. Gender of MEFs used were not determined as these were collected at 15.5 embryonic stage.

Authentication

The identity of MEFs used was authenticated by genotyping the source animal.

Mycoplasma contamination	All cell lines tested negative for Mycoplasma contamination prior to use in experiments.
Commonly misidentified lines (See ICLAC register)	N/A

Animals and other research organisms

Policy information about studies involving animals; ARRIVE guidelines recommended for reporting animal research, and Sex and Gender in Research

Laboratory animals	Mus musculus. The mutant animal line, LYST-B6 (C57Bl/6J-Lystem1-40mal), was generated by electroporation of CRISPR/Cas9 guide RNA to zygotes and surgically implanted int pseudo-pregnant recipient CB6F1 female mice. Founders were screened by PCR and Sanger sequencing and backcrossed to WT C57BL/6J. Mutant colony was then maintained in a homozygous state.
Wild animals	The C57Bl/6J strain (#000664) was obtained from Jackson Laboratory, Bar Harbor, ME, USA.
Reporting on sex	All adult animal studies for behavioral studies, bleeding assays, platelet analysis, histological analysis, and ~omics studies utilized male mice because of resource limitation. For molecular validation, including measurement of gene specific RNA expression, equal numbers of male and female mice was utilized. Detailed gender and numbers include the following:<br><br>Experiments were performed at various ages<br>Genotyping PCR: 3-month-old mice; 1 mouse per genotype<br>qPCR: 3-month-old mice; 6 animals per strain (3 males and 3 females)<br>Western blotting: 3-month-old-mice<br>IHC cerebellum: 18-month-old mice<br>IF cerebellum: 3-, 6-, and 12-month-old mice<br>EM nerve fibrils: 3-month-old and 24-month-old mice; 3-5 animals per age/strain<br>EM platelets: 9-month-old mice<br>Blood smears: 6-month-old mice<br>Bleeding assay: 3-month-old male mice; 5 animals per strain<br>Behavioral: 10 age groups from 6- 15 months of age; 5-21 animals per sex/strain group<br>• 6 mo: KO- 7 M + 10 F ; WT- 9 M +10 F<br>• 7 mo: KO- 7 M + 10 F ; WT- 9 M +10 F<br>• 8 mo: KO- 7 M + 10 F ; WT- 9 M +10 F<br>• 9 mo: KO- 9 M + 17 F; WT- 5 M + 16 F<br>• 10 mo: KO- 6 M + 11 F; WT- 9 M + 10 F<br>• 11 mo: KO- 6 M + 11 F; WT- 9 M + 8 F<br>• 12 mo: KO- 11 M + 10 F; WT- 17 M + 14 F<br>• 13 mo: KO- 6 M ; WT- 9 M<br>• 14 mo: KO- 6 M ; WT- 9 M<br>• 15 mo: KO- 6 M ; WT- 9 M<br><br>Lipidomics: 18-month-old male mice; 3 animals per strain<br>Transcriptomics: 3-month-old and 18-month-old male mice; 3 animals per age/strain
Field-collected samples	N/A
Ethics oversight	Animal studies were done under an approved study protocol, G-14-3, and follow National Institutes of Health and National Human Genome Research Institut e Animal Care Use Committee (Bethesda, MD, USA) approved guidelines. Animals were held in an AAALAC-approved Facility.

Note that full information on the approval of the study protocol must also be provided in the manuscript.

Plants

Seed stocks	N/A
Novel plant genotypes	N/A
Authentication	N/A
